# In vivo chromatin organization on native yeast telomeric regions is independent of a cis-telomere loopback conformation

**DOI:** 10.1186/s13072-020-00344-w

**Published:** 2020-05-22

**Authors:** Emeline Pasquier, Raymund J. Wellinger

**Affiliations:** Department of Microbiology and Infectious Diseases, Faculty of Medicine and Health Sciences, Université de Sherbrooke, Cancer Research Pavilion, Rm 3025, 3201, rue Jean-Mignault, Sherbrooke, QC J1E 4K8 Canada

**Keywords:** Telomeric chromatin, Telomere, Subtelomere

## Abstract

**Background:**

DNA packaging into chromatin regulates all DNA-related processes and at chromosomal ends could affect both essential functions of telomeres: protection against DNA damage response and telomere replication. Despite this primordial role of chromatin, little is known about chromatin organization, and in particular about nucleosome positioning on unmodified subtelomere–telomere junctions in *Saccharomyces cerevisiae.*

**Results:**

By ChEC experiments and indirect end-labeling, we characterized nucleosome positioning as well as specialized protein–DNA associations on most subtelomere–telomere junctions present in budding yeast. The results show that there is a relatively large nucleosome-free region at chromosome ends. Despite the absence of sequence homologies between the two major classes of subtelomere–telomere junctions (i.e.: Y’-telomeres and X-telomeres), all analyzed subtelomere–telomere junctions show a terminal nucleosome-free region just distally from the known Rap1-covered telomeric repeats. Moreover, previous evidence suggested a telomeric chromatin fold-back structure onto subtelomeric areas that supposedly was implicated in chromosome end protection. The in vivo ChEC method used herein in conjunction with several proteins in a natural context revealed no evidence for such structures in bulk chromatin.

**Conclusions:**

Our study allows a structural definition of the chromatin found at chromosome ends in budding yeast. This definition, derived with direct in vivo approaches, includes a terminal area that is free of nucleosomes, certain positioned nucleosomes and conserved DNA-bound protein complexes. This organization of subtelomeric and telomeric areas however does not include a telomeric cis-loopback conformation. We propose that the observations on such fold-back structures may report rare and/or transient associations and not stable or constitutive structures.

## Background

Telomeres, the ends of linear chromosomes, are specialized nucleoprotein structures primordial for genome stability. Generally composed of short, tandem repeats of DNA sequences that end in a single-strand extension of the 3′-end, telomeres and associated proteins ensure protection of natural DNA ends and avoid them being recognized as sites of damage [1]. The telomere proximal area, also called subtelomeric area, usually is gene-poor and enriched in complex repeated elements of various structures [2]. This overall organization of the terminal areas of chromosomes is highly conserved in eukaryotes, from yeast to mammals. *Saccharomyces cerevisiae* telomeres comprise of 300 ± 75 bp of heterogenous telomeric repeats (abbreviated (TG_2-3_(TG)_1-6_)n, hereafter also referred to as TG-repeats) and recruit numerous proteins [3]. This terminal repeat-containing area appears devoid of nucleosomes and instead is covered by multiple Rap1 molecules that recruit Rif1, Rif2 and the SIR complex (Sir2, Sir3, Sir4) [3–5].

Yeast subtelomeric repeat elements, i.e., X elements and Y’ elements, influence several aspects of telomere functions such as telomere length regulation and transcriptional repression induced by telomeres, also known as Telomere Position Effect (TPE) [6–8]. Y’ elements exist in two forms (Y’ long and Y’ short) and are found in 1 to 4 copies at approximately half of chromosomal ends [9, 10]. Unlike Y’ elements, X elements are found at all chromosomal ends and are much more heterogenous in length and sequence [10]. On chromosomal ends with both the X and Y’ elements, the X element is found on the centromeric side of the Y’ element and in certain instances, there are several telomeric repeats at the X–Y’ junction [3]. Despite the fact that there is no detectable sequence homology between X and Y’ elements, most subtelomere–telomere junctions share binding sites for several DNA-binding proteins such as the ORC complex, Tbf1 and Reb1 [2, 11–13]. The ORC complex functions in replication origin firing and silencing, and binds DNA at ARS consensus sequences (ACS) [14–17]. Tbf1 and Reb1 proteins are sequence-specific DNA-binding factors with numerous genomic-binding locations in vivo, mainly in gene promoter regions [18–22]. It is thought that they act on chromatin organization by allowing proper nucleosome positioning on either side of their binding location [18, 23, 24]. In addition to those binding sites above, X elements also harbour an Abf1 binding site [2]. Like Reb1, Tbf1 and Rap1, Abf1 is a General Regulatory Factor (GRF), an abundant and essential protein with numerous DNA-binding sites at gene promoters [23, 24].

On subtelomeric areas, chromatin organization such as nucleosome positioning has been investigated in small and large scale. However, direct analyses for nucleosome positioning over X elements via MNase experiments so far have been reported only for one unmodified chromosomal end (TEL03L) [25]. Other analyses concerned modified chromosomal ends with the *URA3* or *ADE2* genes inserted upstream of the X element [26]. However, it has become clear that these marker insertions and analyses for TPE for example may be difficult [27]. Also, marker insertion at any location in the genome can affect the transcriptional potential of an area surrounding the marker and hence the chromatin configuration may not reflect that of the native state [28]. Genome wide analyses of nucleosome positioning suggested a low content of nucleosomes across the X elements [29], whereas a parallel study suggested positioned nucleosomes over those same areas [30].

A particularity of subtelomeric chromatin is that the SIR complex appears enriched over telomeric repeats and X elements [29, 31, 32]. SIR-bound chromatin at subtelomeres or at mating-type loci is refractory to transcription, and also to other DNA-related processes like replication origin firing or DNA repair [33]. Upon recruitment of Sir2 via the concerted action of DNA-bound Rap1, the ORC complex and Abf1, it deacetylates histones of nearby nucleosomes allowing Sir3 and Sir4 binding to hypoacetylated histones, eventually leading to SIR complex propagation to adjacent chromatin [33]. On the other hand, at chromosome ends, bound Tbf1 and Reb1 at subtelomeres appear to counteract this SIR complex spreading [34].

Compelling results suggest that due to multiple interactions between themselves and histones, the SIR proteins may facilitate a chromatin folding, resulting in specific high-order structures. ChIP analyses on cross-linked chromatin showed that Rap1 is not only bound to the terminal telomeric repeats, as expected, but it unexpectedly is also associated with subtelomeric sites relatively far from *bona fide* Rap1-binding sites [35, 36]. The results also showed that this association is SIR dependent. Moreover, experiments using a reporter construct, in which an Upstream Activating Sequence (UAS, or enhancer) was positioned downstream of the coding region, allowed the detection of an SIR-dependent transcription of the reporter [37]. These findings lead to the suggestion that terminal telomeric repeats fold back onto subtelomeric areas, hence allowing the UAS to relocate upstream of the gene and activate transcription of it [37]. The SIR-dependent transcription of the reporter gene with the downstream UAS is only genetically detectable when the construct is inserted close to telomeric repeats and does not occur when it is inserted elsewhere in the genome [37]. The contacts of terminal Rap1 proteins with subtelomeric areas can be explained with a similar logic [35, 36] and this cis-telomere fold-back model therefore is also congruent with the Rap1 enrichment over X elements, even in an X–Y’ context where the X-element is separated from the physical end by more than 5 kb [29]. Finally, this high-order structure at telomeres is thought to participate in TPE and the protection of chromosomal ends from ectopic recombination [8, 38–40].

Here, we used the in vivo ChEC method to characterize subtelomeric chromatin organization in its native state, without any inserted sequence or marker gene nearby. We analyzed a number of distinct subtelomere–telomere junctions and report a detailed account of similarities and differences in chromatin organization between Y’-telomeres and two X-telomere junctions (TEL03L and TEL06R). Our detailed molecular analyses on X–Y’ junctions that are far from the physical chromosome end on which they reside are incompatible with the classical view of the cis-telomere fold-back model. We suggest that the dynamic behavior of telomeric areas may have allowed the previous observations, but that for most of the time, the constitutive telomeric chromatin does not involve a loop-back structure.

## Results

### In vivo ChEC analyses of the chromatin organization at Y’-telomere junctions

The in vivo ChEC method (for Chromatin Endogenous Cleavage), based on fusions of specific DNA-binding proteins of interest with the catalytic domain of micrococcal nuclease (MNase), was initially developed and applied to map the DNA-binding sites of the certain proteins on genomic sequences [41–43]. In short, MNase-dependent DNA cuts are induced in permeabilized yeast cells in vivo by addition of calcium ions. The localization of the induced cleavages by diverse techniques, i.e., Southern blot or high-throughput sequencing, allows the identification of high-affinity binding sites of the protein of interest. On the other hand, purified MNase has been used to detect protein-bound DNA sequences, for example nucleosomal DNA in vitro [44]. In this way, MNase has been used extensively to analyse protein positioning on DNA, and as in the case mentioned, nucleosome positioning. We reasoned that identification of MNase-protection patterns on DNA by performing ChEC experiments with expressed nuclear MNase could provide not previously available information on in vivo chromatin organization. To overexpress free nuclear MNase, we expressed it fused to a nuclear localization sequence (NLS) from the inducible gal1-10 promoter, used as control in previous studies [41, 42]. We also overexpressed MNase fused to the Gal4 DNA-binding domain (GBD). Given the absence of known Gal4-binding sites in our regions of interest, i.e., subtelomeres and telomeres, we similarly consider this GBD-MN a non-targeted nuclear protein. In addition, we analyzed MNase-dependent DNA cuts induced by MN-Rap1 [42, 43]. Rap1 being an abundant telomeric protein, it will yield a high local concentration of MNase at telomeres and provide a demarcation point for the transition between telomeric repeats bound by Rap1 and subtelomeric sequences [43]. Finally, to map subtelomeric nucleosomes on the same regions, we used a previously developed H2A-MN construct [41, 45]. A limitation of the ChEC method is that on areas with multiple closely spaced potential cut sites, conclusions on the dynamics of individual sites are difficult to draw. Also, it may not always be possible to know whether a cut is caused by a bound or an unbound fused protein [42].

**Fig. 1:**
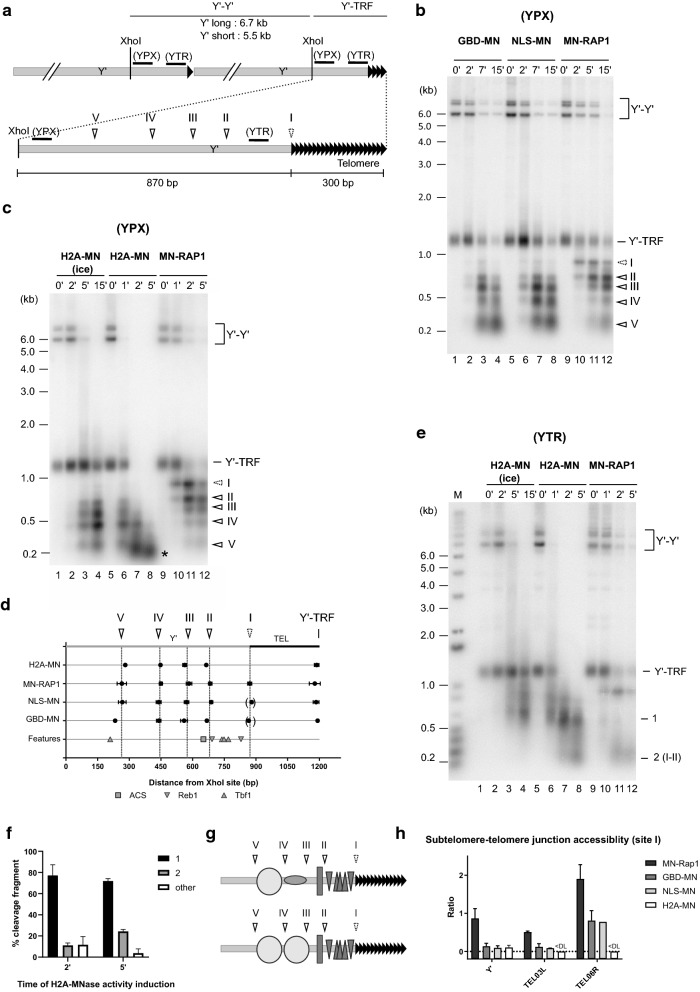
Chromatin organization of Y’ elements. **a** Schematic drawing of Y’ elements with position of the conserved XhoI site. Y’-specific probes (YPX, YTR) are indicated by a solid black line. TRF: Terminal Restriction Fragment. Distal black triangles represent terminal repeat sequences. MNase-fused protein-induced cutting on terminal fragments (Y’-TRF), as derived from **b** is indicated by arrowheads. Solid line arrowheads show observable cutting common to all MNase-fused proteins, whereas the dotted line arrowhead corresponds to cutting mainly observable with MN-RAP1. **b** In vivo ChEC experiments with GBD-MN (W3749-1a + pRSE), NLS-MN (W3749-1a + pG1NLS2), and MN-RAP1 (EPY007) as analyzed on Y’ elements. Southern blot with XhoI-digested genomic DNA hybridized to the YPX Y’-specific probe. Time of MNase activity in minutes is indicated on top of gel. Arrowheads as in **a**. DNA marker sizes are indicated on left of gel (kb). **c** In vivo ChEC experiments with H2A-MN (EPY130) and MN-RAP1 (EPY007) analyzed at Y’ elements. Gel annotations as in **b.** *: end-point mono-nucleosomal fragment (see Additional File 1: Fig. S1c). **d** Location of MNase-sensitive sites on Y’-TRF with known features indicated. Averaged fragment sizes ± standard deviation of MNase-induced cutting determined from at least 2 independent experiments are plotted with respect to the XhoI site. Location of features (Y’ element, ACS, and Tbf1 and Reb1 binding sites) from a representative Y’-TRF sequence, TEL08L, is included. Parentheses around cutting sites indicate extremely low cutting efficiency that could not be quantified consistently. **e** In vivo ChEC experiments with H2A-MN (EPY130), and MN-RAP1 (EPY007) analyzed at Y’ elements. The blot shown in **d** was re-hybridized with the more distal YTR Y’-specific probe that encompasses Tbf1- and Reb1-binding sites (see **a**). M: DNA size marker. **f** Quantification of the fraction of fragments 1 and 2 (see gel in **e**) generated by H2A-MNase (30 °C). Average percent ± standard deviation from 2 independent ChEC experiments is plotted. **g** Model of chromatin organization of Y’ terminal fragments. Nucleosomes are depicted by circles, the ORC complex by a rectangle and Tbf1/Reb1 proteins by triangles. **h** Accessibility of Y’-telomere junctions and X-telomere junctions to MNase-fused proteins. Ratio of cleavage products detected at the Y’-junction (% signal from band I) on cleavage products detected around Y’-ACS (% signal from band II and III) for each MNase-fused protein. Ratio of cleavage products detected at the X-junction (% signal from band I) on cleavage products detected around X-ACS (% signal from band V and VI for X(03L)-telomere, and from band III for X(06R)-telomere for each MN-fused protein. DL: Detection Limit

We first focused on Y’ elements, representing approximately half of all yeast chromosomal ends (Additional file 1: Fig. S1a; [10]). First, we mapped MNase-dependent cut sites reflecting MNase accessible DNA sites on Y’-terminal restriction fragments (Y’-TRF) by Southern blotting with a Y’-specific probe proximal to the XhoI site that is conserved on all Y’ elements (YPX probe) (Fig. 1a, b). The combination of results obtained with MN-Rap1, GBD-MN and NLS-MN yielded five MNase-dependent cut sites in the Y’-TRF region analysed (indicated by arrowheads in Fig. 1b–d). Cleavage efficiencies of MNase-sensitive sites varied depending on the specific MN-fused protein and will be discussed below. Unexpectedly, spacing between some of these MNase-dependent cuts were inconsistent with the presence of phased nucleosomes upstream of telomeres, as was suggested in previous studies [4, 29]. Two MNase-protected DNA regions, between cutting sites II and III (106 ± 15 bp) and between III and IV (129 ± 11 bp), are significantly shorter than the 146 bp expected and minimally required in the case of phased nucleosomes (Fig. 1d and Additional file 1: Fig. S1b). Furthermore, between the first two MNase-dependent cuts (cutting sites I and II) is an area with a high density of binding sites for the Tbf1 and Reb1 proteins, two general regulatory factors (Fig. 1d and Additional file 1: Fig. S1a). If all those sites were bound by the cognate proteins, the first approximately 200 bp of the Y’ element proximal to telomeric repeats could be devoid of nucleosomes. To test this hypothesis, we fused MN to a canonical histone core protein, H2A, and determined the in vivo ChEC-induced cleavage pattern in this area. The experiment was performed on ice (4 °C) or at 30 °C, which allowed an assessment of cleavages occurring with different digestion kinetics (Additional file 1: Fig. S1c, d). The very partial H2A-MN cleavage at 4 °C indeed is necessary to detect MNase-cutting sites that are distant from the probe used. Ca^2+^-induced H2A-MN cleavage on ice during 15 min resulted in a partial digestion with visible mono-, di-, tri-, and tetra-nucleosome sized DNA fragments as assessed by whole genomic DNA analysis (Additional file 1: Fig. S1c lane 4). A comparable extent of cleavage is observed after 1 min of Ca^2+^-induced H2A-MN at 30 °C (Additional file 1: Fig. S1c lane 6). The resulting cleavage pattern at 4 °C analyzed with the YPX probe is very similar to that induced by GBD-MN and NLS-MN (compare Fig. 1c left with Fig. 1b). However and remarkably, while cleavage at cut site I is very efficient by the MN-Rap1 protein (Fig. 1b right), it is undetectable in the H2A-MN strain and only very weakly observable with the GBD-MN and NLS-MN proteins (Fig. 1b, c, dotted line arrowhead). This result thus is consistent with an absence of a nucleosome proximal to telomeric repeats. The complete H2A-MN digestion obtained after 5 min of Ca^2+^-induced H2A-MN at 30 °C allowed a direct assessment of whether there was a nucleosome in this area. Southern analysis with the YPX probe shows only mononucleosome-sized fragments near the probe (Fig. 1c, lane 8 (*)) similar to the ethidium bromide stain for bulk chromatin (Additional file 1: Fig. S1c, lane 8). In contrast, Southern analysis with a probe complementary to the stretch of Tbf1- and Reb1-binding sites on the Y’-TRF (YTR probe, Fig. 1a, e) yielded only a very low signal for the I–II fragment, annotated 2 on Fig. 1e and most of the signal remained in a smear near 600 bp that corresponds to the terminal fragment from site II to the end (annotated 1, Fig. 1e, lane 8), inconsistent with positioning of a nucleosome. Indeed, quantification of the gels indicates that only 24.3 ± 1.8% of the total signal corresponds to the I–II fragment after 5 min of Ca^2+^-induced H2A-MN at 30 °C (Fig. 1e, f). These results suggest two possible arrangements for chromatin on terminal Y’-sequences as depicted in Fig. 1g. In both models, the 200 bp of Y’ sequences proximal to the terminal repeats (DNA between cut sites I and II) are not occupied by a nucleosome. Most likely, these sequences are bound by Tbf1 and Reb1 proteins. In addition, the next short protected area between sites II and III covers the verified Y’-ACS and thus could be bound by the origin recognition complex (ORC, see discussion below for a more complete description).

As mentioned above, the subtelomere–telomere junction appeared to be differentially accessible to MN-fused proteins. A band corresponding to a cleavage at this position (MNase dependent cut site I) appeared early in the Ca^2+^-induced MN-Rap1 time course (2 min) and is the main cleavage product (Fig. 1b, c). To estimate and compare MNase accessibility to the Y’-telomere junction according to MN-fused proteins, we determined the ratio of cleavage products detected at the Y’-junction (% signal from band I) on cleavage products detected around the Y’-ACS (% signal from bands II and III) for each MN-fused protein (Fig. 1h). In contrast to MN-Rap1 (Ratio cleavage I/II + III: 0.87 ± 0.26), a very low accessibility of this subtelomere–telomere junction cutting site is observed with untargeted nuclear MNases, i.e., GBD-MN, NLS-MN, or H2A-MN (Fig. 1b, c, h) (Ratio cleavage I/II + III: 0.14 ± 0.08 (GBD-MN); 0.09 ± 0.003 (NLS-MN), 0.10 ± 0.06 (H2A-MN)) suggesting an inaccessibility of the Y’-telomere junction to free nuclear proteins and H2A.

The above results confirm the suitability of in vivo ChEC to analyse chromatin organization at subtelomere–telomere junctions. Furthermore, the results strongly suggest that the most distal portion of the subtelomeric regions of telomeres with Y’-elements, i.e., approximately half of the telomeres, are bound by Reb1 and Tbf1 proteins but lack a nucleosome.

### In vivo ChEC analyses for the chromatin organization at X-telomere junctions

**Fig. 2:**
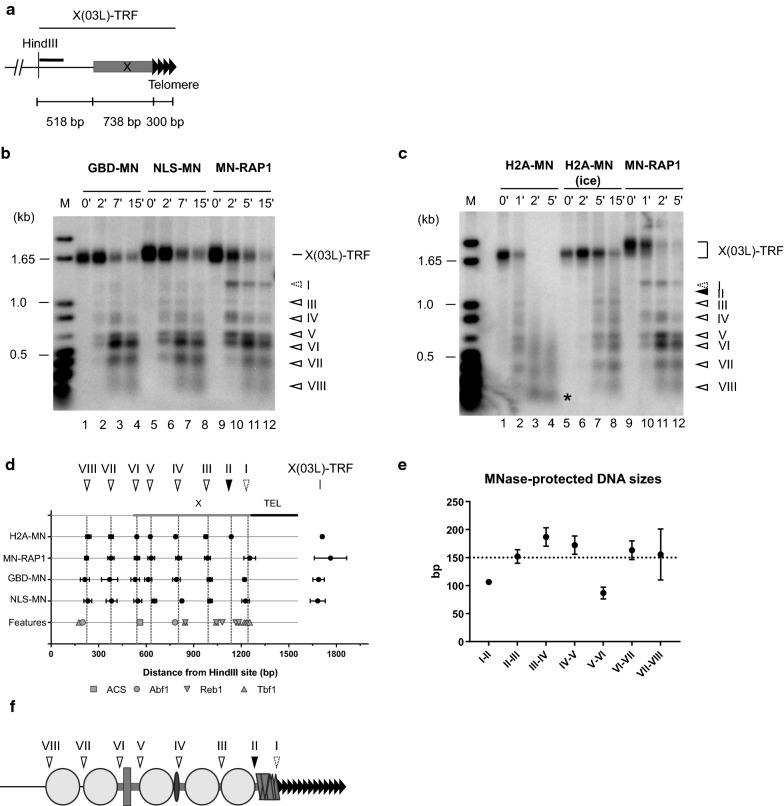
Chromatin organization of the terminal TEL03L X element. **a** Schematic drawing of the TEL03L X element with the location of the HindIII site. The TEL03L-specific probe used in **b** and **c** is represented by a solid black line. TRF: Terminal Restriction Fragment. **b** In vivo ChEC experiments with GBD-MN (W3749-1a + pRSE), NLS-MN (W3749-1a + pG1NLS2), and MN-RAP1 (EPY007) analyzed on TEL03L. Southern blot with HindIII-digested genomic DNA hybridized to the TEL03L-specific probe. Time of MNase activity in minutes is indicated on top of gels. Solid line arrowheads show observable cutting common to all MNase-fused proteins, whereas the dotted line arrowhead corresponds to cutting observable predominately with MN-RAP1. M: DNA size marker with DNA marker sizes indicated on left of gel (kb). **c** Same as in **b** with H2A-MN (EPY130) and MN-RAP1 (EPY007) strains. The solid black arrowhead corresponds to very weak cutting observable with H2A-MN. **d** Location of MNase-sensitive sites on TEL03L with known features associated. Averaged fragment sizes ± standard deviation of MNase-fused protein-induced cutting determined from at least 2 independent experiments is plotted with respect to the HindIII site. Location of features (X-element, ACS, and Abf1, Tbf1- and Reb1-binding sites) is included. **e** Average distance ± standard deviation between MNase-sensitive sites identified in Fig. 2b, c. Size greater than 146 bp is congruent with positioning of a nucleosome, indicated by a dotted line. **f** Model of chromatin organization of TEL03L terminal fragment. Symbols as in Fig. 1, with addition of Abf1 as an oval

We applied the same procedures to analyse the chromatin organization of two X-telomere junctions: those at the TEL03L and TEL06R chromosomal ends. A TEL03L-specific probe identified eight MNase-dependent cut sites on the TEL03L-TRF (Fig. 2a–c). Two MNase-protected DNA regions, namely those observed between cutting sites I and II (approximately 106 bp) and between cutting sites V and VI (87 ± 10 bp), are too short to be able to accommodate a positioned nucleosome (Fig. 2d, e). Interestingly, between cutting sites I and II, a high density of potential Tbf1- and Reb1-binding sites is found, suggesting that the last 100 bp of the TEL03L-X element is protected by bound Tbf1 and Reb1 (Fig. 2d). Note that in strains without additional mutations as in Fig. 2, the MNase-cutting site II is detected only with H2A-MN and is relatively weak (black arrow on Fig. 2c), but it is readily detectable in strains with mutations in SIR complex genes (see below). The spacing between cutting sites II and III (152 ± 12 bp) and between III and IV (187 ± 16 bp) being congruent with the size of DNA protected by nucleosomes (Fig. 2e), we favor a model where these areas are associated with nucleosomal particles, despite a few potential Tbf1- and Reb1-binding sites identified (Fig. 2d, f). Interestingly, the cut site IV (average location: 804 ± 21 bp from the HindIII site) is close to an Abf1-binding site (centered at 783 bp from the HindIII site), suggesting that at this telomere, an MNase-sensitive DNA area is induced by Abf1 bound to its cognate site (Fig. 2d). The results also suggest a nucleosome positioned upstream of the Abf1-binding site (between MNase-sensitive sites IV and V) and ORC binding on the TEL03L-ACS (between cutting sites V and VI) (Fig. 2d, f). With complete H2A-MN digestion, we observed mostly mono-nucleosome size fragments (*) (Fig. 2c lane 4) as expected with the presence of nucleosomal arrays close to the probe used.

These results suggest that as shown above for the Y’-telomere junction, the region proximal to the TG repeats of X-only telomeres is devoid of nucleosomes. This hypothesis was assessed by Southern blotting H2A-MN ChEC samples with a telomeric probe (Additional file 1: Fig. S2). Note that this probe will reveal all telomeric bands, the X-only and Y’-telomeres at the same time. In the case of a positioned nucleosome abutting the terminal telomeric TG repeats and complete Ca^2+^-induced H2A-MN digestion, we expected the appearance of a smear centered on the average TG repeat length (300 ± 75 bp). However, the experiment yielded two distinct smears, one centered approximately at 600 bp (annotated 1 on the gels of Additional file 1: Fig. S2) and the other one approximately at 450 bp (annotated 3 on the same gels, Additional file 1: Fig. S2 lane 8). In contrast, when using MN-Rap1 and an induction time of 5 min, the expected smear close to 300 bp indeed became detectable (annotated 4 on Additional file 1: Fig. S2, left, lane 12). These findings are inconsistent with a nucleosome positioned next to TG-repeats on any chromosome end.

**Fig. 3:**
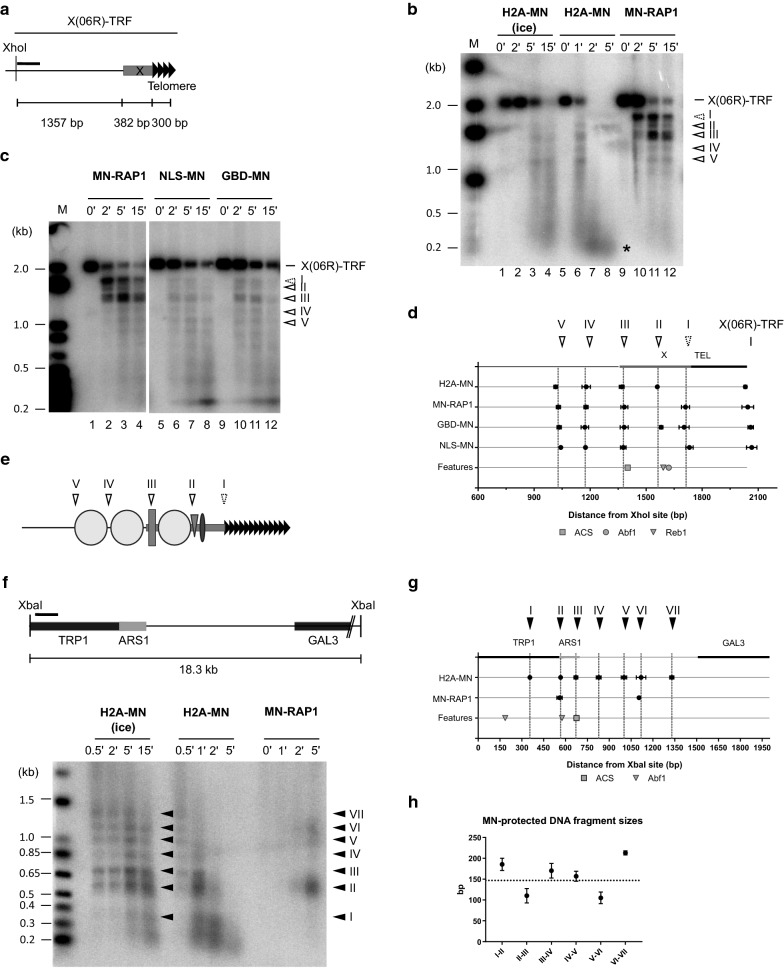
Chromatin organization of the terminal TEL06R X element. **a** Schematic drawing of TEL06R X element with the position of XhoI site. The TEL06R-specific probe used in **b** and **c** is represented by a solid black line. **b** In vivo ChEC experiments with H2A-MN (EPY130) and MN-RAP1 (EPY007) analyzed on TEL06R. Southern blot with XhoI-digested genomic DNA hybridized with a TEL06R-specific probe. Arrowheads with a solid line show detectable cutting common to all MNase-fused proteins, whereas the stippled arrowhead corresponds to cutting not observed with H2A-MN. M: DNA size marker with DNA marker sizes indicated on left of gel (kb). **c** Same as in **b** with GBD-MN (W3749-1a + pRSE), NLS-MN (W3749-1a + pG1NLS2), and MN-RAP1 (EPY007) strains. **d** Location of MNase-sensitive sites on TEL06R with known features associated. Averaged fragment sizes ± standard deviation of MNase-induced cutting determined from at least 2 independent experiments is plotted with respect of XhoI site. Location of features (X-element, ACS, and Abf1, Tbf1 and Reb1 binding sites) is included. **e** Model of chromatin organization of TEL06R terminal fragment. Symbols as in Fig. 1. **f** Top: schematic of the analyzed *TRP1ARS1* locus on chromosome IV. Short black bar indicates the probe used. Below: in vivo ChEC experiments with H2A-MN (EPY130) and MN-RAP1 (EPY007) analyzed on *TRP1ARS1* locus. Southern blot with XbaI digested genomic DNA hybridized with a *TRP1*-specific probe. Solid black arrowheads correspond to cutting observable with H2A-MN. DNA marker sizes are indicated on left of gel (kb). **g** Schematic diagram of the detected MN-cut sites from **f**. Labeling is as in Fig. 1d. **h** Average distance ± standard deviation between MNase-sensitive sites identified in **g**

Given that the non-nucleosomal regions in Y’ or X(TEL03L) upstream of the telomeric repeats show potential binding sites for Tbf1 and Reb1 (Fig. 1c, d), we decided to analyse an X-only telomere, TEL06R, that lacks such potential binding sites for Tbf1 and Reb1 proximal to the TG repeats (see Additional file 1: Fig. S3a). ChEC analysis as above with a TEL06R-specific probe identified five preferential MNase-dependent cut sites on the TEL06R-TRF (Fig. 3a–d). Except for the fragment between cut sites I and II (spacing of 143 ± 9 bp), all MNase-protected DNA fragments were larger than 146 bp, arguing for positioned nucleosomes on those fragments (Additional file 1: Fig. S3b). Mono-nucleosome sized fragments (*) visible with complete H2A-MN digestion (Fig. 3b) confirm nucleosome arrays close to the probe used. As for the X(TEL03L)-telomere junction, we did not detect an H2A-MNase-dependent cut at the X(TEL06R)-telomere junction (MNase cut site I, Fig. 3b). Moreover, on X(TEL06R) the DNA region between MNase cut site I and II appears to be poorly protected from being cut by MNase. Indeed, with GBD-MN or MN-Rap1, a non-negligible diffuse cutting signal is observed between these two preferential MNase cut sites if compared to MN-Rap1-induced cuts in the corresponding area of TEL03L (Fig. 3c, Additional file 1: Fig. S3c). In specific, after 5 min of Ca^2+^-induced GBD-MNase or MN-Rap1, the bands corresponding to preferential MNase cut sites I and II on TEL06R are almost completely replaced by a very diffuse signal (Fig. 3c, Additional file 1: Fig. S3c). On TEL03L, a 5-min induction of MN-Rap1 still yields a sharp band with virtually no background cutting on either side (Additional file 1: Fig. S3c).

Moreover, the X(TEL06R)-telomere junction appears to be more accessible to free MNase than the X(TEL03L)-telomere junction (Fig. 1h). As previously discussed, to compare MNase accessibility to the subtelomere–telomere junction, we determined the ratio of cleavage detected at cut site I (subtelomere–telomere junction) versus cleavages detected around the X-ACS (Cleavages V and VI for X(TEL03L)-telomere, and cleavage III for X(TEL06R)-telomere). Compared to TEL03L or a Y’ telomere, this ratio increased sevenfold (GBD-MN) and ninefold (NLS-MN) for TEL06R (Fig. 1h). This site is also more accessible for MN-Rap1, but the relative increase is slightly less dramatic (about 3.5-fold increase for TEL06R as compared to TEL03L, Fig. 1h).

These results argue that on TEL06R, proximal to the TG-repeats, there is an area of increased MNase accessibility and that is potentially bound only by one Reb1 protein and one Abf1 protein. By extension, here again there may be no nucleosome abutting the TG repeats. From these observations, we propose a model of the chromatin organization of X(TEL06R)-TRF, depicted in Fig. 3e, which despite the clear differences of this telomeric X compared to other telomeric X elements, resembles a general model for X- and Y’-telomeres.

A common thread of all the findings reported to here on the terminal sequences of Y’- and X-telomeres is a strong and general MN-sensitivity very close to the ACS sequences, which could be attributed to ORC-binding (see Figs. 1b–g, 2b–f and 3b–e). To verify whether these observed patterns here are consistent with the well-established chromatin organization on a known ACS locus near an ARS sequences or particular for subtelomeric elements, we analyzed the *TRP1ARS1* locus near centromere IV in an analogous fashion (Fig. 3f–h). The *ARS1* locus contains well-documented ACSs and probably is the origin of replication with the most detailed information on chromatin organization and ORC binding (for example, [46, 47]). Hence, it is well established that ORC binds to this ACS and causes a nucleosome-free region starting in G2 all the way to the next S-phase [47]. The analyses of that locus with our technology are very consistent with these findings: a nucleosome-free area on the ACS region (cut site II to cut site III in Fig. 3f, g), and this area is accessible for cutting by free MN-Rap1 late in the time-course (Fig. 3f). Left and right from that short fragment are strongly positioned nucleosomes (Fig. 3f), as expected from ORC binding [47]. These characteristics therefore very closely parallel the results for the ACS sites on the X- as well the Y’-telomeres (Figs. 1, 2, 3). Altogether, these results strongly suggest that the sequences around the subtelomeric ACSs are ORC-bound, nucleosome-free fragments.

### Chromatin organization at the subtelomere–telomere junctions is independent of telomere length and SIR2

**Fig. 4:**
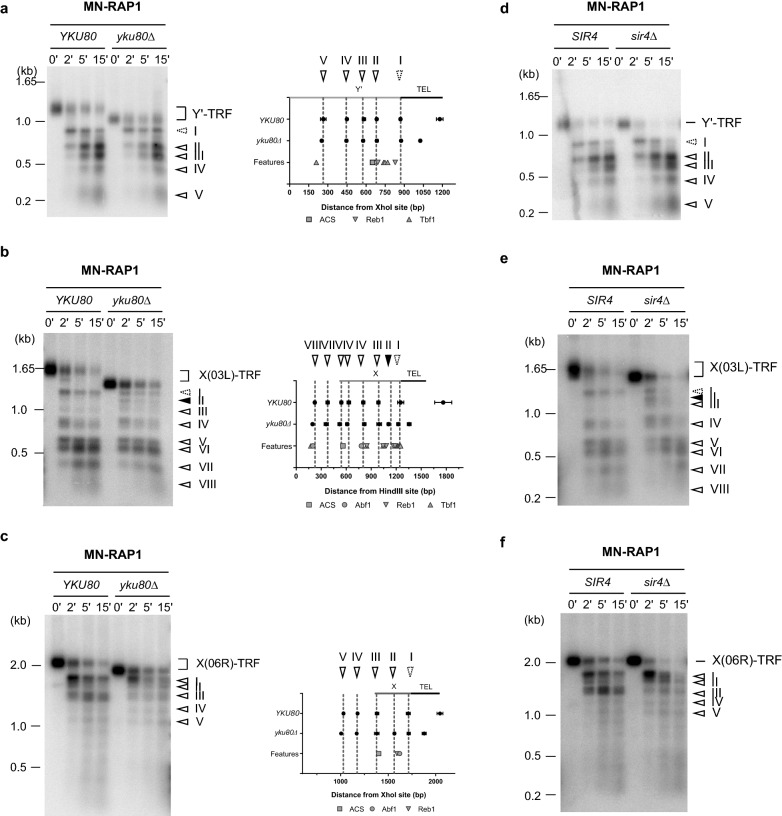
Chromatin organization of subtelomeric elements is independent of telomere length and the SIR complex. In vivo ChEC experiments with MN-RAP1 analyzed on Y’ elements in **a**, TEL03L in **b** and TEL06R in **c** in presence (*YKU80*) or in absence of the Yku complex (*yku80Δ*). In each panel, the left shows a Southern blot with digested genomic DNA hybridized with the appropriate specific probe. On the right is a schematic diagram with the averaged MN-Rap1-induced cutting sites ± standard deviation determined from at least 2 independent experiments plotted with respect to the XhoI site and other previously described features. **d**–**f** In vivo ChEC experiments with MN-RAP1 analyzed on Y’ elements in **d**, TEL03L in **e** and TEL06R in **f** in presence (*SIR4*) or in absence of the SIR complex (*sir4Δ*)

To test if short telomeres induce changes in chromatin organization at subtelomere–telomere junctions, we analyzed MN-Rap1 ChEC patterns in *yku80Δ* cells of the three subtelomere–telomere junctions analyzed above. In addition to a short telomere phenotype and altered physical DNA ends, *yku80Δ* cells also suffer from telomere capping defects and a loss of TPE [48–52]. Despite expecting a lower amount of MN-Rap1 proteins at telomeres, MN-Rap1 ChEC patterns for the Y’-TRFs were virtually identical *in YKU80* and *yku80Δ* cells (Fig. 4a and Additional file 1: Fig. S4a). Similarly, all previously identified MNase-sensitive sites for TEL03L-TRF were identical in *YKU80* and *yku80Δ* cells (Fig. 2b, c and 4b). Remarkably, the previously determined MNase cut site II detected only with H2A-MN in wt cells becomes readily detectable with MN-Rap1 in *yku80Δ* cells (Fig. 4b and Additional file 1: Fig. S4b). These observations suggest that on TEL03L too, the absence of the Yku complex has no impact on the location of MNase-protected DNA fragments despite increased accessibility of the previously described MNase-sensitive site II (black arrow in Fig. 4b and Additional file 1: Fig. S4b). In a similar manner, we observed that MNase-sensitive site locations remained unchanged between *YKU80* and *yku80Δ* cells when analysing TEL06R-TRF (Fig. 4c).

In addition, we tested if SIR2-dependent histone tail deacetylation is implicated in the chromatin organization at the subtelomere–telomere junctions. Sir2 being recruited to telomeres via an Sir4-mediated interaction with Rap1, we analyzed MN-Rap1 ChEC pattern in *sir4Δ* cells [15, 35]. Overall, MN-Rap1 ChEC patterns obtained on DNA in *sir4Δ* cells are indistinguishable from the ones obtained in *yku80Δ* cells. For example, the MN-Rap1 ChEC pattern in the absence of the Sir4 protein is unchanged for Y’-TRF (Fig. 4d). For TEL03L-TRF and TEL06R-TRF, in *sir4Δ* cells, we detected MNase cut sites at the previously characterized MNase-sensitive sites (Fig. 4e, f). Moreover, as detected in *yku80Δ* cells, in *sir4Δ* cells we observed an increase in accessibility of MNase cutting site II to MN-Rap1 for the TEL03L X-telomere junction (Fig. 4e).

We conclude from the above results that the locations of MNase-sensitive sites and, by extension, the locations of nucleosomes and proteins bound to subtelomeres are independent of telomere length and Sir2-dependent histone tail deacetylation.

### Chromatin organization at internal X–Y’ junctions analyzed by in vivo ChEC

**Fig. 5:**
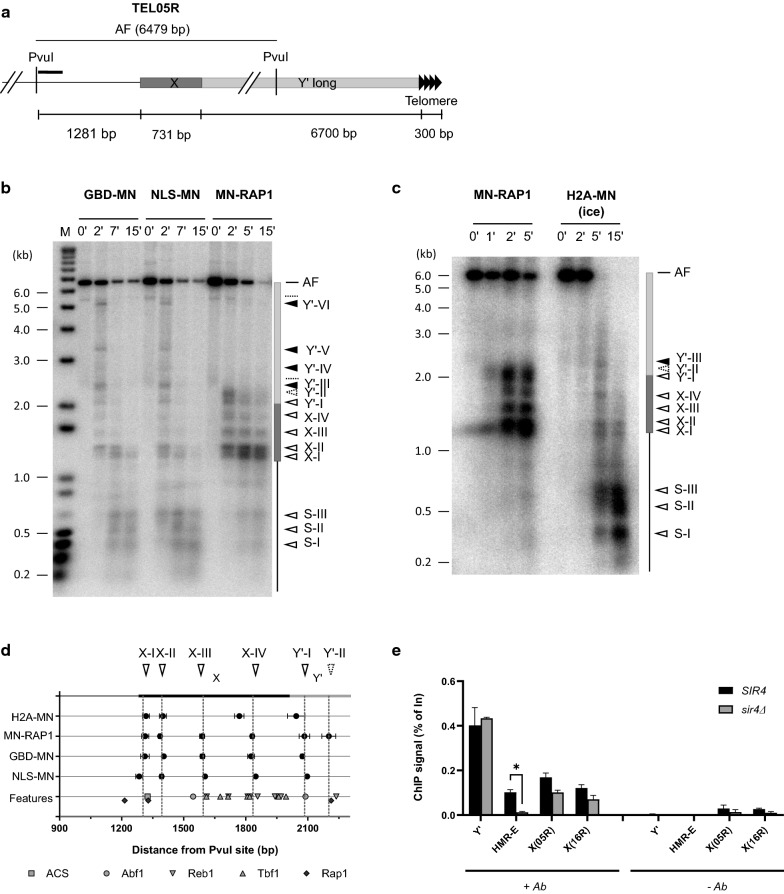
Chromatin organization of the X element on TEL05R (XY’ context). **a** Schematic drawing of the X–Y’ junction area on TEL05R with the positions of the PvuI sites. The TEL05R-specific probe used in **b** is represented by a solid black line. AF: Analyzed Fragment. **b** In vivo ChEC experiments with GBD-MN, NLS-MN and MN-RAP1 analyzed on the TEL05R X–Y’ junction fragment. Southern blot with PvuI-digested genomic DNA hybridized to the TEL05R-specific probe. Time of MNase activity in minutes is indicated on top of gel. Arrowheads with solid line show detectable cutting common to all MNase-fused proteins, whereas the arrowhead with dotted line corresponds to cutting observable predominately with MN-RAP1, and black arrowheads indicate cutting not detected with MN-RAP1. Band labelled AF is the full-length fragment, and dotted lines indicate two very weak cross-hybridizing non-specific bands. M: DNA size marker with DNA marker sizes indicated on left of gel (kb). **c** Same as in **b** with H2A-MN and MN-Rap1 strains. **d** Averaged fragment sizes ± standard deviation of MNase-induced cuttings from indicated MN-fused proteins determined from at least 2 independent experiments is plotted with respect to the telomere distal PvuI site. Location of features (ACS, Abf1, Tbf1 and Reb1 potential binding sites) is included. Only cutting sites on X element (X–I, X–II, X–III, X–IV) and Y’–I and Y’–II are displayed. **e** Rap1 ChIP analysis on Y’, HMRE, X(05R)–Y’ junction (X(05R)) and X(16R)–Y’ junction (X(16R)). Percent of input DNA recovered from ChIP performed with anti-Rap1 antibody (+ Ab) or without (− Ab) from *SIR4* (W3749-1a) or *sir4Δ* (EPY031) strains. Data are mean ± standard deviation from two biological duplicates. *P value < 0.05 (unpaired t-test). Note that the differences of IP values between the *SIR4* vs *sir4Δ* strains on the X(05R)–Y’ and X(16R)–Y’ loci are not significant

Given the subtelomeric organization of the X- and Y’-elements (see introduction), an X-element can abut either on a terminal TG repeat tract, such as on telomeres TEL03L and TEL06R, or abut to the distal end of a Y’-element. We thus wondered whether the chromatin organization on an X-telomere junction was different of that on an X–Y’ junction. Thus, we analyzed the ChEC pattern of two X–Y’ junctions, those in TEL05R and TEL16R. Whereas some X–Y’ junctions show TG repeats between the X element and Y’ [2], we confirmed by sequencing that, as expected from the available data on published databases, these two X–Y’ junctions do not have TG repeats between the X and Y’ element. However, these X–Y’ junctions show very distinct differences in size, spacing between the X-ACS and the X-Abf1 sites and in the number of potential Tbf1- and Reb1-binding sites (Additional file 1: Fig. S5a). The X element on TEL05R shows an organization and features similar to most X–Y’ areas with a 221-bp spacing between the X-ACS and the Abf1 sites and ends distally in a relatively high density of potential Tbf1- and Reb1-binding sites (Additional file 1: Fig. S5a). Note that overall, the X(TEL05R) is also similar to the X(TEL03L) previously analyzed in the X-only context (Additional file 1: Fig. S3a). To analyze the X–Y’ region of TEL05R by the ChEC method, we used a probe complementary to a subtelomeric region located at 1281 bp from the start of the X element (Fig. 5a). The analyzed fragment (AF) corresponding to this probe is obtained by genomic DNA digestion with PvuI and in addition to the X element, encompasses the first 4467 bp of a Y’(long) element (Fig. 5a). Upstream of the X element start we identified three MNase-sensitive sites (S-I, S-II and S-III on Fig. 5b, c). Moreover, we identified four MNase-sensitive sites on the X(TEL05R) element that are cut by all MN-fused proteins tested: GBD-MN, NLS-MN, H2A-MN and MN-Rap1 (Fig. 5b, c). As previously observed for the X(TEL03L)-TRF, two MNase-sensitive sites flank the X(TEL05R)-ACS located at 1325 bp from the PvuI site, generating a very short protected area of about 85 bp only (X-I to X-II in data summary in Fig. 5d). The cut site X-III centered at 1594 ± 19 bp from the PvuI site is close to the X-Abf1 site (1545 bp from PvuI site). Interestingly, we detected six preferential MNase-sensitive sites on Y’(TEL05R): one shared by all MN-fused proteins tested (cut site Y’-I), one detected only with MN-Rap1 (cut site Y’-II), and four detected with GBD-MN and NLS-MN only (see Fig. 5d). The cut site Y’-I is located at 70 ± 20 bp from the X–Y’ junction and is correlated with a potential Abf1-binding site centered at 75 bp from XY’ junction. The efficient cleavages over the X element with MN-Rap1 (Fig. 5c) strongly suggest a relatively high local concentration of MN-Rap1 over X–Y’ junctions.

The X(TEL16R) shows a spacing between the X-ACS and X-Abf1 site of 192 bp, shorter than the one of X(TEL05R) and with only one potential Tbf1-binding site within the X element (Additional file 1: Fig. S5a). In fact, X(TEL16R) is quite similar to X(TEL06R) which ends at telomeric repeats (see above, Additional file 1: Fig. S3a). According to published sequences, TEL16R harbours a X–Y’(short) junction. It should be noted that we found that in our strains (W303 background), TEL16R shows two consecutive Y’ elements (scheme depicted in Additional file 1: Fig. S5c). Nonetheless, we sequenced the XY’ junction and found no point mutations compared to the sequence from the standard S288C strain background. With our probe hybridizing at 1306 bp from the X element start, and genomic digestion of ChEC samples with BamHI and XhoI, we identified six MNase-sensitive sites (Additional file 1: Fig. S5d, e). Non-specific bands are observed with this probe (dotted line in Additional file 1: Fig. S5d), but the intensity of these bands again is negligible compared to intensity of the band corresponding to our fragments of interest (AF in Additional file 1: Fig. S5c, d). Three of the detected cut sites are located in the subtelomeric region upstream of the X element start site, two are located over the X element and one in Y’. Overall, these MNase-sensitive sites on the X(TEL16R) junction fragment are very similar to those described above for the X(TEL06R) element (see Additional file 1: Fig. S5e). The Y’-I cut site detected with GBD-MN and NLS-MN is located at 65 ± 4 bp from the X–Y’ junction. These data highlight the involvement of X-ACS and Abf1-bound DNA in chromatin organization of X elements, irrespective of whether the X element is terminal or not.

Again and as for the TEL05R above, while no TG repeats with potential Rap1-binding sites are present at the TEL16R X–Y’ junction, a remarkably efficient cutting by MN-Rap1 of the X-element sequences is observed. Detailed sequence analyses revealed certain potential Rap1-binding elements on both of these X-elements (Fig. 5d, Additional file 1: Fig. S5b, e). The efficient MN-Rap1 cutting at these sites on internal X-elements therefore could be due to direct Rap1 binding. We tested this prediction by ChIP experiments (Fig. 5e). Indeed, we could detect a specific Rap1 signal for both X-elements on TEL05R and on TEL16R by ChIP. As expected, a strong signal was also found for terminal Y’-sequences and the single Rap1 bound at the HMR-E element (Fig. 5e) [53]. These results therefore strongly suggest that Rap1 binds directly on X-elements, even on non-terminal ones such as those on TEL05R and TEL16R.

### No cis-telomere fold-back detected by in vivo ChEC

**Fig. 6:**
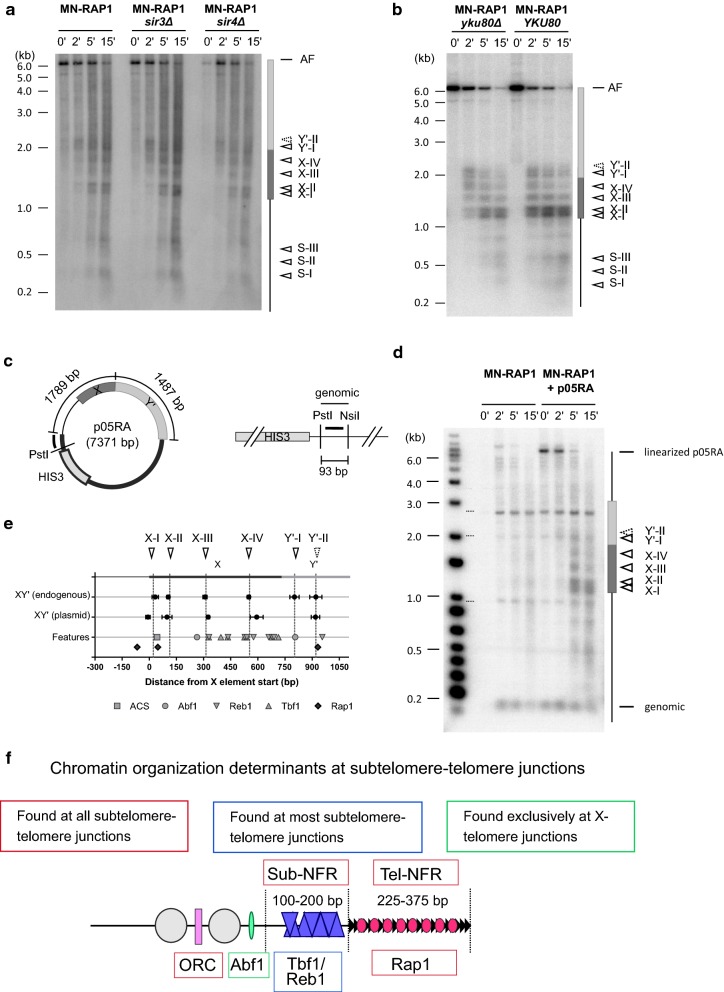
No evidence of a stable telomere foldback structure by in vivo ChEC. **a** In vivo ChEC analysis of the PvuI fragment of TEL05R (as in Fig. 5b) obtained with MN-Rap1 in WT cells (EPY007), *sir3Δ* cells (EPY131) and *sir4Δ* cells (MVL054). **b** Same as in **a** but analysis obtained with MN-Rap1 from WT (EPY007) and *yku80Δ* cells (EPY070). **c** Schematic drawing of the circular plasmid (p05RA) encompassing the TEL05R XY’ junction. Thick line corresponds to plasmid backbone whereas thin line corresponds to TEL05R XY’ junction sequences. The probe used in **d** is depicted by a solid black line next to the PstI site. This probe also hybridizes the genomic HIS3 locus depicted on the right. **d** In vivo ChEC experiments with MN-RAP1 (EPY007) and MN-Rap1 + p05RA, analyzed with the probe indicated in **c**. The Southern blot contained PstI- and NsiI-digested genomic DNA. Time of MNase activity in minutes is indicated on top of gel. MNase-induced cutting is indicated by arrowheads (solid line: cutting previously identified in Fig. 5b as common to all MNase-fused proteins; arrowhead with dotted line corresponds the previously identified cutting specific to MN-RAP1 in the same blot) **e** Location of MNase-sensitive sites on the TEL05R XY’ junction fragment for the endogenous locus (top) and on the circular plasmid (middle). The position of features (ACS, Abf1, Tbf1, Reb1 and Rap1 potential binding sites) is included on bottom. **f** Determinants of chromatin organization at budding yeast subtelomere–telomere junctions

Given the readily detectable MN-Rap1-mediated cut sites as well as direct Rap1 binding in the above two internal X-elements, we tested for the presence or absence of a telomere fold-back structure [29, 35, 37–40]. The model predicts that the distal telomeric TG repeat tract folds back and associates with the internal X-element, bringing the terminal Rap1 molecules in close contact with the X-sequences. This “telomere fold-back model” is supported by several indirect experiments but has not yet been demonstrated directly [35–38, 40]. In an X–Y’ context, the telomere foldback loops out the entire Y’ element in order for the terminal repeat tract to associate with the X-element [29]. However, previous experiments also showed that this cis-telomere foldback is strictly dependent on the SIR complex, i.e., the observed telomere-X interactions were lost in the absence of the Sir proteins [35, 36]. These findings predicted that if the MN-Rap1-dependent cuts detected over the X-element in an X–Y’ context are due to the canonical foldback mechanism, they will be dependent on the SIR proteins. Unexpectedly, the ChEC patterns on the X(05R)–Y’ junction obtained in *sir3Δ* and *sir4Δ* cells are indistinguishable from those obtained with cells that are SIR^+^ (Fig. 6a). In addition, as established by ChIP, the direct Rap1 binding at these X-elements also is independent from the Sir proteins (Fig. 5e), just as the binding of Rap1 on sequences at the ends of Y’-elements. Note that a Sir-dependent Rap1 binding was detectable in our experiments for the HMR-E element (Fig. 5e). Moreover, the yKU complex is also thought to be implicated in the telomere foldback structure [36, 39, 40]. However, our results show an extremely similar MN-Rap1 ChEC pattern of the X(05R)–Y’ junction in *yku80Δ* and *YKU80* cells (Fig. 6b). In contrast and as expected [43, 54], the efficiency of MN cuts over X(05R)-Y’ is significantly decreased in *sir4Δ* when ChEC patterns obtained from strains expressing the Yku70-MN fused protein were analyzed (Additional file 1: Fig. S6a). We conclude that the MN-Rap1-mediated cleavages on the X-element in an X–Y’ context are independent of the SIR and yKU complexes and that Rap1 does bind to these elements directly in a Sir-independent fashion.

The telomere foldback model also predicts that cleavages on an X element analyzed with MN-Rap1 would be dependent on the presence of telomeric repeats on the same DNA molecule. Therefore, we analyzed the MN-Rap1 ChEC pattern on a replicative circular plasmid encompassing the X–Y’ junction from the same TEL05R as above (Fig. 6c, d). In the case of a telomere foldback, we expected a loss of MN-Rap1-induced cleavages over the X element on the plasmid. In contrast, we observed four MNase cut sites over the X element in both endogenous and plasmid context (X–I, X–II, X–III, X–IV on Figs. 5b, d and 6d, e). Comparison of the localization of these cleavages with respect to the X-element start at the endogenous site or the plasmid X(05R)–Y’ junction (Fig. 6e) shows a very analogous cutting pattern with no significant variation of the localization of MN-Rap1-dependent cuts over the X-element in an X–Y’ junction context. At the endogenous locus, two cleavages were detected in the first 200 bp of the Y’ element from TEL05R chromosomal end (Y’–I and Y’–II on Fig. 5b, d). On the p05RA plasmid, the cut Y’-II is observed whereas the cut Y’-I is confounded with a non-specific band (dotted line, Fig. 6d). Nevertheless, we conclude that preferential cuts induced by MN-Rap1 over an X-element in an X–Y’ context are independent of the presence of TG repeats or a physical DNA end on the same DNA molecule.

## Discussion

Here, we analyzed the chromatin organization of several *S. cerevisiae* subtelomere–telomere junctions by in vivo ChEC experiments. It is well established that in budding yeast, the area just proximal to the canonical telomeric repeats is gene-poor and the sequences found there can be classified into two broad classes: ends with a Y’-element at the junction and ends with an X-element at the junction. While the Y’-elements generally are well conserved, the X-elements vary considerably (see Additional file 1: Figs. S1a, S3a). Despite these clear sequence differences at the subtelomere to telomere junction, strikingly our study here suggests that certain specific features are common to all telomeres: (i) a 100 to 200 bp area right next to the telomeric TG repeats is held free of nucleosome binding; (ii) binding of the ORC complex and the Abf1 protein also prevents nucleosome binding and positions them on either side of them; (iii) between those sites and telomere distal of them reside well-positioned nucleosomes; (iv) an absence of nucleosomes on the terminal repeat DNA, a confirmation of previous suggestions [4, 5]. Finally, our analyses of the chromatin architecture at X–Y’ junctions did not reveal any evidence for a stable telomeric loop-back structure.

### Tbf1 and Reb1 at subtelomeres and the subtelomeric nucleosome-free region

Tbf1 and Reb1 binding on predicted binding sites on subtelomeric repeat elements (X and Y’) has been demonstrated in vitro [11–13] and confirmed in vivo [18, 21]. The high number and density of potential binding sites at the telomere proximal side of all the subtelomeric repeats render an assessment of which individual specific site is occupied very difficult in vivo.

On the 7 Y’-telomere junctions that are entirely sequenced (Additional file 1: Fig. S1a), two potential Reb1-binding sites, spaced by 137 bp, are surrounded by 3 to 4 Tbf1-binding sites. The MNase-protected fragment between the nearest two preferential MNase cut sites at these Y’-telomere junctions (i.e., between cut sites I and II) in principle is large enough to accommodate a positioned nucleosome. However, Tbf1 binding on this area of the Y’-TRF has been confirmed by ChIP-seq experiments [18] and evidence of in vivo Reb1 binding on this region is also documented (Additional file 1: Fig. S6b, [21]). While it has been suggested that Reb1-bound DNA could be associated with nucleosomes [22], our results presented in Fig. 1 suggest that this is not the case at Y’-telomere junctions. First, MNase tethered to a nucleosomal protein with the H2A-MN construct appears to have almost no access to the Y’-telomere junction (site I, Fig. 1c, g). Given that these Y’-telomere junctions are highly accessible to MN-Rap1 (Fig. 1b, c), we exclude that the inaccessibility of these junctions to H2A-MN is due to intrinsic MNase properties. Second, the H2A-MN ChEC analysis with the YTR probe (region encompassing the Tbf1- and Reb1-binding sites) is incompatible with a positioned nucleosome on this region (compare Fig. 1c lane 8, e lane 8). We therefore conclude that this area proximal to the telomeric repeat sequences is not occupied by a nucleosome but rather by Tbf1 and Reb1 proteins.

On X elements, the organization of potential binding sites for Tbf1 and Reb1 is less conserved. In most cases, several Tbf1-binding sites (2 to 5) are located at less than 50 bp from X–Y’ or X-telomere junctions followed by two to three Reb1-binding sites located at less than 100 bp from these junctions (X-only telomeres: TEL13R, TEL10R, TEL14R, TEL02R, TEL04L, TEL09R, TEL07L, TEL11L, TEL01L, TEL03L, TEL15L, TEL03R, TEL11R, X–Y’ chromosomal ends: TEL02L, TEL13L, TEL12R, TEL14L, TEL12L, TEL06L). More distal to the telomere, the area located at 100 bp to 400 bp from the junctions also shows potential-binding sites for Tbf1 and Reb1 (4 to 5 for Reb1, 2 to 3 for Tbf1). However, this stretch of distal Tbf1-binding sites is absent at several XY’ junctions (TEL04R, TEL05R, TEL08R, TEL07R, TEL15R, TEL16L), and a few X elements from XY’ junctions also show an absence of the distal Reb1-binding stretch (TEL10L, TEL09L, TEL08L, TEL16R, TEL05L). Two exceptional X-telomere junctions (TEL01L and TEL06R) show few or no potential Tbf1- and Reb1-binding sites. Our experiments target a majority class X-telomere (TEL03L), an exception (TEL06R, see Additional file 1: Fig. S3a), as well as the majority class X–Y’ junction (TEL05R) and an exceptional X–Y’ junction (TEL16R; see Additional file 1: Fig. S5a). Remarkably, for both types of terminal X-elements, there is a nucleosome-free area proximal to the X-telomere junction (Fig. 2f, e), which of course also mirrors the situation on all the Y’-telomere junctions (see above, Fig. 1g). Therefore, we propose that a short (100–200 bp) stretch of sequences abutting the terminal telomeric repeats is held free of nucleosomes. At present, mutant alleles of Tbf1 or Reb1 that completely lost DNA binding are not available and experiments to assess chromatin of this area in absence of Tbf1 or Reb1 binding are not feasible.

### The ORC complex, Abf1 and Rap1 in subtelomeric chromatin organization

The ORC complex binds to specific DNA sequences, marks replication origins and participates in establishing a gene-silencing environment [14–17]. The ARS Consensus Sequence (ACS) is absolutely required for ORC complex binding to DNA and is a feature shared by all subtelomere–telomere junctions in *S. cerevisiae.* It has been shown that the ORC complex covers 38–44 bp of DNA in vitro [55]. On non-subtelomeric areas, the ORC-bound ACS is asymmetrically located in a nucleosome-free region of approximately 125 bp and flanked by two well-positioned nucleosomes [47]. In agreement with an ORC-bound ACS on X(TEL03L)-ACS, X(TEL05R)-ACS and Y’-ACS, we observed two MNase-sensitive sites around them spaced respectively by 86.7 ± 10.5 bp, 90.8 ± 18.3 bp, and 106 ± 15.1 bp. On the X(TEL06R)-ACS, the MNase-cutting sites appear even closer and not easily distinguishable, but the gel-tracings of MN-Rap1 and GBD-MN experiments do hint at the presence of two cuts (Additional file 1: Fig. S3c). On the X(TEL16R)-ACS, we observed a relatively broad area being subject to cleavage centered on or close to the X-ACS (cleavage product X-I on the X(TEL16R), Additional file 1: Fig. S5d). While two sites cannot be detected, the broad band at least is compatible with an MNase cutting on either side of an ORC. We therefore hypothesize that the combined ChEC patterns support an ORC-bound DNA on all X and Y’-subtelomeric repeats. The small ACS area would then be held nucleosome free on all these various repeats.

In support of this conclusion, an analogous analysis of the well-defined ACS in the ARS1 locus yielded results that are very consistent with this interpretation (Fig. 3f–h): a short 110-bp fragment next to the ACS that is also accessible to MN-Rap1 late in the time course. It remains possible that ORC binding on subtelomeric ACSs only occurs during part of the cell cycle, but not constitutively. Furthermore, formally, our results cannot exclude the possibility that these ACS-associated short fragments are bound by non-canonical and ‘unstable’ nucleosomes.

An Abf1-binding site is found in all X-elements (Additional file 1: Figs S3a and S5a) and on all X elements analyzed in this study, we observed a relatively broad area surrounding the binding site accessible to MNase (cleavage product IV for X(TEL03L) in Fig. 2, cleavage product II for X(TEL06R) in Fig. 3, cleavage product X-III for X(TEL05R) in Fig. 5, cleavage product X-II for X(TEL16R) in Additional file 1: Fig. S5). Abf1 being able to act as a nucleosome barrier [23, 24], these observations are coherent with Abf1-bound DNA and displaced nucleosomes.

### Well-positioned nucleosomes in the telomere proximal region

Our study supports the idea that telomeres act as a barrier for nucleosome positioning as previously proposed [4, 30]. Indeed, cut site I is observed at X(03L)-TRF and X(06R)-TRF, located respectively at 15.2 and 26.0 bp from the beginning of the TG repeats (Figs. 2d, 3d). A very similar MN-Rap1-specific cut site I also occurs very close to the Y’-telomere junctions (Fig. 1b, d). However, in contrast to the X-telomere junctions, the X–Y’ junctions do not contain an MNase-sensitive site (Fig. 5c and Additional file 1: Fig. S5e). Of note, the analyzed XY’ junctions do not contain remnants of terminal telomeric repeat sequences, while a number of other X–Y’ junctions do (Additional file 1: Fig. S6d). These observations therefore argue for a direct impact of multiple Rap1-bound telomeric repeats and associated proteins on chromatin organization. Moreover, the fact that we failed to detect fragments shorter than 200 bp generated with the MN-Rap1 ChEC experiment as analyzed with a pan-telomeric probe (Smear 4 on Additional file 1: Fig. S2a) suggesting that the protein complexes associated with Rap1-bound TG repeats are compacted and render the underlying telomeric DNA difficult to access by MNase, even to MN-Rap1.

Depending on the specific location of the ACS- and Abf1-binding sites on the various X- or Y’-elements, we propose the occurrence of strongly positioned nucleosomes (see Figs. 1g, 2f, 3e). Furthermore, our results are consistent with positioned nucleosomes telomere distally. Hence, the various telomere-binding complexes and proteins appear to yield a strong nucleosome positioning in this area and this effect is independent of the specific underlying sequence.

The chromatin organization model for the TEL03L-telomere derived from our in vivo ChEC experiments is in full agreement with the one proposed by Vega-Palas working with purified MNase on spheroplasts [25, 56]. It must be noted that these studies analyze chromatin organization in a cell population and the results cannot exclude the possibility that Tbf1 and Reb1 could bind at some other potential binding sites. Also as previously suggested, our results suggest a similar chromatin organization at all X-only telomeres [25, 26]. Indeed, all X-only telomeres show a conserved spacing of 221 ± 1 bp between the X-ACS- and Abf1-binding sites (Additional file 1: Fig. S3a), coherent with a well-positioned nucleosome between the DNA bound by the ORC complex and Abf1. Moreover, a high density of Tbf1- and Reb1-binding sites in the last 100-bp upstream of the TG repeats is shared by most X-only telomeres (Additional file 1: Fig. S3a), suggesting that Tbf1 and Reb1 proteins cap the distal portion of subtelomeric regions (see above).

In summary, the closest nucleosome to the subtelomere–telomere junctions is located at a certain distance from the actual junction, depending on the nature of the subtelomeric repeat element, more than 100 bp from X-telomere junctions, and more than 200 bp from Y’-telomere junctions. Indeed, in the case of the Y’-telomere junctions, the data suggest that the TG-repeat abutting stretch of Tbf1- and Reb1-bound DNA is in a continuum with the ORC-bound ACS, followed by a nucleosome or an unknown protein (Fig. 1g).

### High level of MN-Rap1-induced cleavages on X elements but no evidence of a loop-back structure

We were intrigued by the high efficiency of MN-Rap1-induced cleavages on telomere distal X-elements, i.e., X elements in an X–Y’ context (Fig. 5b, Additional file 1: Fig. S5d). Knowing that no internal telomeric repeats are present at both X–Y’ junctions analyzed (TEL05R and TEL16R; Additional file 1: Fig. S6d), we expected a low level of MN-Rap1 cutting, if any at all, and in contrast to the X elements in an X-only telomere context (TEL03L and TEL06R). Previous results with MN-Rap1 on non-telomeric loci (*HIS4, RPL21A* and *RSP9B* promoter regions) showed that the earliest detected cuts during a time course of Ca^2+^-induced MN-Rap1 cleavage were highly correlated with known Rap1-binding sites [43]. Rap1 being an abundant nuclear protein, some MNase-sensitive sites far from known Rap1-binding sites were also detected, but these were detected much later during the time course. Similar observations were reported with an Rap1-MN ChEC-Seq approach [42]. The early and strong detection of MN-Rap1 cuts over X-elements in an X–Y’ context (see for example 2 min of Ca^2+^-induced MN-Rap1 in Fig. 5b) suggests that the local concentration of MN-Rap1 is high over these telomere distal X-elements. Indeed, analyses of ChIP-seq data revealed evidence of Rap1 binding on X elements [29]. Nevertheless, efficient MN-Rap1 cleavages on X elements could also be explained by a telomere foldback structure that loops out the entire Y’ element and brings Rap1-bound TG repeats close to the X element [29, 35–40]. However, MN-Rap1 induces cleavages on the X-element of the same X–Y’ junction even if this fragment is placed on a circular plasmid without any TG-repeat sequences (Fig. 6d, e). Therefore, MN-Rap1 cleavage over this X element is independent of the presence of TG repeats or a physical DNA end on that same DNA molecule. Moreover, the MN-Rap1-generated ChEC pattern on this telomere distal X does not change in *sir3Δ*, *sir4Δ*, or *yku80Δ* cells (Fig. 6a, b). This despite the fact the SIR complex and yKU complex have been reported to be required for both a telomere foldback structure as well as telomere clustering at the nuclear periphery [35, 37, 39, 40, 57]. These results altogether are incompatible with the idea that a telomere fold-back or other clustering is the source for the observed high-efficiency MN-Rap1 ChEC patterns over the internal X-element. On the other hand, a re-examination of the X-elements via bio-informatic Rap1-binding site predictions identified several hitherto unknown potential binding sites on X elements (Additional file 1: Fig. S6c, d) and ChIP experiments showed that there in fact is direct and SIR-independent Rap1 binding on these X-elements (Fig. 5e). The most straightforward interpretation thus is that the high-efficiency MN-Rap1 cleavages on X elements are due to direct Rap1 binding. It is worth noting that our study is based on ChEC experiments that do not involve protein–DNA crosslinking or PCR-based signal enhancement procedures. Therefore, rare and/or transient interactions between the terminal telomeric proteins and X-associated proteins very likely remain undetected. Indeed, we speculate that clustering of telomeres at the nuclear periphery could allow transient interactions between heterochromatin-like structures, i.e., telomeres, X elements, mating type loci, and such interactions may be detectable by ChIP as SIR- and yKU-dependent Rap1 associations far from the physical chromosomal ends. However, our examination of the bulk telomere distal X-elements shows that such clustering is not stable or frequent enough to yield a detectable signal in Southern blots.

## Conclusions

Altogether, our results highlight that despite no sequence homology between the two major subtelomere–telomere junctions in budding yeast (Y’-telomeres and X-telomeres), chromatin organization similarities are found for all telomeres. A nucleosome-free region just upstream of the TG-repeats is found at all analyzed terminal junctions (Fig. 6f). Furthermore, Tbf1 and Reb1 are often DNA-bound on these subtelomeric nucleosome-free regions. Despite variations in the distance from the junctions, a DNA-bound ORC complex also appears to be common to all subtelomere–telomere junctions. Finally, ORC- and Abf1-induced nucleosome positioning is shared by all X-telomeres and occurs at various distances from the junctions.

While establishing a general framework for the in vivo chromatin organization of all chromosome ends, the results also show that this chromatin organization is independent of a stable cis-telomere foldback structure. Considering the previous results that lead to a telomere foldback model, we suggest that a dynamic behavior of chromosome ends may allow the documentation of transient and/or rare telomere–internal DNA interactions. These latter interactions however would depend on genetic setup and analytic methods used and not be part of the constitutive architecture.

## Methods

### Strains and plasmids

All strains used in this study are derivatives of W303 and genotypes are described in Table 1. To express H2A fused to the catalytic domain of micrococcal nuclease (MN), we constructed the EPY130 strain by a one-step replacement of the HTA1 gene with an HTA1-MN-(HA)_3_::kanMX6 cassette. The HTA1-MN-(HA)_3_::kanMX6 cassette DNA was obtained by PCR from strain JS311H2AMN [45]. To express GBD (Gal4 Binding Domain) or NLS (Nuclear Localization Signal) fused to MN, we used strain W3749-1a transformed respectively with pRSE or pG1NLS2. EPY070 and EPY129 strains were obtained by a PCR-based one-step replacement of the YKU80 gene with respectively an LEU2 cassette and URA3 cassette [58]. Strain EPY031 was obtained by a one-step replacement of the SIR4 gene with a KanMX6 cassette. Strain EPY131 was obtained by a one-step replacement of the SIR3 gene with an HIS3 cassette. Yeast strains obtained were verified by Southern blot. In all experiments, yeast cells were grown at 30 °C with constant agitation in standard conditions (YEP or YC media with appropriate carbon sources).

**Table 1:** Yeast strains used

Strain	Genotype	References
W3749-1a (WT)	*Mat A ade2*-*1 can1*-*100 ura3*-*1 his3*-*11,15 trp1*-*1 leu2*-*3,112 bar1∆::LEU2*	[59]
EPY007 (MN-RAP1)	*Mat alpha ade2*-*1 can1*-*100 his3*-*11,15 trp1*-*1 leu2*-*3,112 MN*-*L1*-*Rap1 ura3*-*1 (L1:linker1)*	[43]
JS311H2AMN	*Mat alpha his3*-*200 leu2*-*1 met15*-*0 trp1*-*63 ura3*-*167 RDN1::Ty1*-*MET15 RDN1::mURA3*-*HIS3 Hta1*-*MN*-*(HA)3::kanMX6*	[45]
EPY130 (H2A-MN)	*Mat A ade2*-*1 can1*-*100 ura3*-*1 his3*-*11,15 trp1*-*1 leu2*-*3,112 bar1∆::LEU2 Hta1*-*MN*-*(HA)3::kanMX6*	This study
MVL054	EPY007 +* sir4Δ::kanMX4*	[43]
EPY070	EPY007 + *yku80Δ::LEU2*	This study
EPY031	W3749-1a + *sir4Δ::kanMX4*	This study
EPY129	W3749-1a + *yku80∆::URA3*	This study
EPY131	EPY007 + *sir3Δ::HIS3*	This study
MVY221 (yKU70-MN)	*Mat A Yku70*-*MN::TRP1 ade2*-*1 ura3*-*1 his3*-*11,15 leu2*-*3,112 trp1*-*1 can1*-*100*	[43]
MVL047	MVY221 +* bar1Δ::natMX4 *+* sir4Δ::kanMX4*	[43]

Plasmids are described in Table 2. pUGM2 was obtained by inserting the Gal1-10prom-GBD-MN from pRSE [43] into the SacI-KpnI sites of pRS316. A region of the pUGM2 backbone was deleted by a one-step site-directed deletion using pRSdel_F and pRSdel_R primers leading to pGM1 plasmid (primers provided in Table 3). To construct plasmid pG1NLS2 that allows expression of an NLS-MN fused protein via the Gal1-10 promoter, a PCR site-directed mutagenesis was done with NLS-MN-F and NLS-MN-R primers on plasmid pGM1 to replace the GBD sequence by the NLS sequence. Plasmid p05RA was constructed by inserting the XY’ region from TEL05R into the NsiI-SalI sites of pRS313. The inserted XY’ region from TEL05R encompasses 1058 bp upstream of the X element, the entire X element and the first 1487 bp of Y’ element. This insert was obtained by a PstI-SalI digestion of a PCR product from W3749-1a genomic DNA using primers described in Table 3. All plasmids obtained were verified by sequencing. The genomic sequences of the internal X–Y’ junctions were from -584 to +989 bp with respect to X element start for TEL05R, and from -655 to +1120 bp for TEL16R.

**Table 2:** Plasmids used

Plasmids	Description (Backbone)	Reference
pRSE	*TRP1, CEN, Gal1*-*10prom*-*GBD*-*MN* (pRS314)	[43]
pUGM2	*URA3, CEN, Gal1*-*10prom*-*GBD*-*MN* (pRS316)	This study
pGM1	*URA3, CEN, del4881*-*142, Gal1*-*10prom*-*GBD*-*MN* (pRS316)	This study
pG1NLS2	*URA3, CEN, del4881*-*142, Gal1*-*10prom*-*NLS*-*MN* (pRS316)	This study
p05RA	*HIS3, CEN, XY’(from TEL05R)* (pRS313)	This study

**Table 3:** Oligonucleotides used

Name	Sequence (5′ to 3′)	Purpose (description)
pRSdel_F	TAACTATGCGGCATCAGAGC	Cloning (Deletion of part of pRS plasmid, del4881-142)
pRSdel_R	GGGCCTCGTGATACGCCT	
NLS_MN_F	TTTCTTTGGCGGCATCCTGCAGCCCGGG	Cloning (Replacement of GBD sequence with NLS sequence)
NLS_MN_R	AAGAGAAAGGTGAAAGGTCAAAGACAGTTGACTGTATCG	
TEL05R_F (specific to TEL05R)	ACGATCGCGTCATTTTACAATG	Cloning (Amplification of XY’ from TEL05R)
TEL05R_YP_R (non specific to TEL05R)	ACCTGATCATGCAATTAGCAAGC	
TEL03L_F	AGCTTTCATCATTCGCGCTGA	Probing (DNA fragment specific for TEL03L)
TEL03L_R	CGTCAACAGGTTATGAGCCCT	
TEL06R_F	CATGAGTTCGAGTATGGTGTT	Probing (DNA fragment specific for TEL06R)
TEL06R_R	GCATGATGATCCCCAATAAC	
TEL05R_F	ACGATCGCGTCATTTTACAATG	Probing (DNA fragment specific for TEL05R)
TEL05R_R	GCAGTCCTTTTGGTCAAAACC	
Y’_XS_F	TGGAGTTTTTCAGCGTTTGCG	Probing (DNA fragment hybridizing in Y’, downstream of XhoI site, YPX probe)
Y’_XS_R	ATCAGCATCGACAGGAATGCC	
Y’_TR_F	TGAAAATGAAACCCTGTTCTTTAGC	Probing (DNA fragment hybridizing in Y’, encompassing Tbf1 and Reb1 binding sites, YTR probe) qPCR (Primer pair used to determine ChIP signal on Y’)
Y’_TR_R	AACAGGGCTTGGAGGAGA	
p05RA_oligo	TGCATTACCTTGTCATCTTCAG	Probing (Oligonucleotide used to probe p05RA plasmid)
TRP1_F	CCGATGCTGACTTGCTGGG	Probing (DNA fragment specific for TRP1 locus)
TRP1_R	TGCCGTAATCATTGACCAGAGCC	
HMR-E 3f	CGAACGATCCCCGTCCAAGTTATG	qPCR (Primer pair used to determine ChIP signal on HMRE [53])
HMR-R 2r	TCGGAATCGAGAATCTTCGTAATGC	
X(05R)Y_F	GGGTTGGTGGTAGGAAGTAGAGGG	qPCR (Primer pair used to determine ChIP signal on X(05R)-Y’ junction)
X(05R)Y_R	GCAATAAGGTGACATAGATATGCTATCCTAATC	
X(16R)Y_F	GTGTGGAATATGAAAGTAGGGTAAGTTTGAGATG	qPCR (Primer pair used to determine ChIP signal on X(16R)-Y’ junction)
X(16R)Y_R	TCGAAGTAAAGGAGCCTACCACTC	

### In vivo ChEC

Cells were pre-grown in YC minimal media with 2% raffinose. Strains endogenously expressing MN-fused proteins (H2A-MN, MN-RAP1, yKU70-MN) were grown in YC complete, strains transformed with pRSE (GBD-MN) in YC-TRP, the ones transformed with pG1NLS2 (NLS-MN) in YC-URA and the ones transformed with p05RA in YC-HIS. To induce GBD-MN or NLS-MN expression in logarithmically growing cells (OD_660_ of 0.5-0.6), galactose was added to a final concentration of 2% for 1 h. To avoid any effects of the carbon source on the ChEC assay, galactose was also added for 1 h to logarithmically growing cells expressing H2A-MN or MN-RAP1 under endogenous promoters. ChEC assays were performed on 100 mL of growing cells as previously described [41, 43]. In brief, cells were harvested and washed in 2 mL of A-PBPi buffer three times. Cells were then resuspended in 1.2 mL of Ag-PBPi buffer containing 1% of digitonin and incubated at 30 °C for 5 min to permeabilized cells. MN activity was induced by addition to Ca^2+^ to a final concentration of 2 mM. Aliquots were taken at indicated time points after addition of Ca^2+^ and MN activity was stopped with 2X STOP solution (400 mM NaCl; 20 mM EDTA; 4 mM EGTA; 0.2 μg/μl glycogen).

### DNA isolation and Southern blotting

ChEC samples were transferred to glass tubes with an equivalent volume of glass beads. Cells were mechanically disrupted by 9 cycles of 30 s of vigorous vortexing followed by 30 s resting on ice. Cell lysates were recovered and transferred to microtubes. DNA extraction was done using a standard phenol–chloroform procedure [60]. An appropriate quantity of digested DNA, ranging from 0.5 µg to 1.5 µg, was separated on 0.75% TBE (1 x) agarose gels, transferred on a Hybond-XL nylon membrane (Amersham) and hybridized to specific ^32^P-labelled radioactive probes. Specific probes to one chromosomal end, to Y’ or to *TRP1ARS1* locus were obtained by genomic DNA PCR-based amplification using specific primers followed by amplicon purification on gel and random priming labeling procedure (Feinberg and Vogelstein, 1983). Sequences of primers used to amplify a specific chromosomal end, a region of Y’ or *TRP1ARS1* locus are depicted in Table 3. As a telomeric repeats probe, a 300-bp fragment containing 280-bp TG repeats derived from pYLPV was radiolabelled using a random priming labeling procedure. A radioactive single-strand oligonucleotide probe hybridizing in p05RA plasmid was obtained by 5′-end-labelling [60] (Table 3). Data were visualized with a Typhoon FLA9000 apparatus. When necessary, membranes were stripped with boiling 0.1% SDS and left at room temperature for 30 min and rehybridized with other radioactive probes.

### DATA analysis

GelAnalyzer software was used to determine the size of each detectable fragment on ChEC-Southern blots according to MNase induction time. Fragment sizes for each MNase induction time and for each MN-fused protein were averaged to determine MNase-sensitive site locations ± Standard Deviation. Distances between MNase-sensitive sites were determined for each ChEC-Southern blot lane. The distance obtained between consecutive MNase-sensitive sites, regardless of MN-fused protein analyzed, was averaged to determine MNase-protected fragment sizes ± Standard Deviation.

Band intensities of each detectable fragment on ChEC-Southern blots were quantified with ImageJ software [61]. The percentage of each fragment signal with respect to total lane signal was calculated. The percentage of one fragment or intensity ratio between fragments was averaged ± Standard Deviation from two independent experiments for H2A-MN, three independent experiments for GBD-MN and NLS-MN, and four independent experiments for MN-Rap1. Plot profiles were derived from ImageJ analysis of each ChEC-Southern blot lane. Pixel intensity according to migration distance for each lane was determined and visualized as a percent of total lane signal.

To identify potential protein-binding site locations on DNA, the software RSAT (Regulatory Sequence Analysis Tools) was used [62] with matrix profiles from JASPAR database (Rap1: MA0359.1, Tbf1: MA0403.1, Reb1: MA0363.1, Abf1: MA0265.1). To include locations of ACS, a position weight matrix of ORC complex binding site was used [46, 63]. To filter potential binding sites, we used a P value < 0.001 for Rap1, Reb1 and Abf1, P value < 0.005 for Tbf1 and P value < 0.000001 for ACS.

Graphics were generated using GraphPad Prism version 8.2.1 for Windows, GraphPad Software, San Diego, California USA, http://www.graphpad.com.

### Chromatin Immunoprecipitation

200-ml cultures were grown at 30 °C to OD600 0.5-0.8 in YEPD. Cells were cross-linked with 1% formaldehyde for 20 min, agitating every 5 min. The cross-linking reaction was stopped by incubation in 125 mM of glycine for 5 min. The culture was split into 50-ml aliquots and washed twice with cold TBS (20 mM Tris–HCl pH 7.5, 150 mM NaCl). Cells were resuspended in 250 µl ChIP lysis buffer (50 mM HEPES–KOH pH 7.5, 140 mM NaCl, 1 mM EDTA pH 8.0, 1% Triton X-100, 0.1% Na-deoxycholate, 1 mM PMSF) containing 1× Complete Mini Protease Inhibitor (Roche). Cells were lysed with 250 µl of glass beads using a FastPrep (MP Biomedicals) 4× 30 s at 6.5 m/s, with 2-min rest on ice in between cycles. Lysate was collected, 400 µl lysis buffer was added and the mix was sonicated (Misonix ultrasonic liquid processor) for 25 cycles (amplitude 100, 1 min on/1 min off on ice), and then centrifuged at 4 °C for 10 min at 14,000 rpm, conserving the supernatant (WCE). 500 µl WCE was incubated overnight rotating at 4 °C with 5 µl 0.4 µg/µl Rap1 (Y-300) sc-20167 rabbit polyclonal IgG (Santa Cruz Biotechnology). 20 µl WCE was incubated overnight at 4 °C without antibody (input). 50-µl equilibrated Pierce Protein A/G Magnetic Beads (#88802) were added for 1 h for chromatin immunoprecipitation. ChIP beads were subjected to the following 1-ml 4-min washes with collection on a magnet between washes: 2X lysis buffer without protease inhibitor, 2× lysis buffer with [50 mM] NaCl, 2× wash buffer (10 mM Tris–HCl pH 8.0, 250 mM LiCl, 0.5% IGEPAL CA630, 0.5% Na-deoxycholate, 1 mM EDTA pH 8.0), 1X TE pH 8.0. The IP was eluted with 50 µl TE pH 8.0 + 0.1% SDS at 65 °C with 12,000 rpm agitation for 10 min, vortexing after 5 min. A second 5-min elution was done with 150 µl TE/SDS. 180 µl TE pH 8.0 + 0.1% SDS was added to the input. After overnight crosslink reversal at 65 °C, the samples were incubated for 1 h at 37 °C with 195 µl TE-SDS 0.1%, 3 µl 10 mg/ml RNaseA, and 2 µl 20 mg/ml glycogen. 7.5 µl 10 mg/ml Proteinase K and 11 µl SDS 10% were added and incubated for 1 h at 50 °C. DNA was extracted using a phenol/chloroform (2X) and chloroform (1×) method and precipitated at − 20 °C overnight with 200 mM NaCl and 2 volumes cold 100% EtOH. Pellets were washed in 70% EtOH and resuspended in 50 µl H_2_0. Immunoprecipitated and input DNAs were diluted and subjected to qPCR using indicated primers and analyzed by the % input method.

## Supplementary information

**Additional file 1.** Additional figures.

## Data Availability

All data generated or analysed during this study are included in this published article and its additional files.
