# Supplementary material for: In vivo chromatin organization on native yeast telomeric regions is independent of a cis-telomere loopback conformation
Source: Epigenetics Chromatin. 2020 May 22;13:23. doi: 10.1186/s13072-020-00344-w (PMC7243337; doi:10.1186/s13072-020-00344-w)
Supplement: Supplementary file 1 — Additional file 1. Additional figures. [file 13072_2020_344_MOESM1_ESM.pdf]

Additional File 1
for

E. Pasquier and R. J. Wellinger

In vivo chromatin organization on native yeast telomeric regions is independent of a cis-telomere loopback conformation.

- 6 Supplemental Figures
- Legends to the Supplemental Figures

Fig. S1

Chromatin organization of terminal Y' elements. a Features identified on terminal portions of Y' elements, starting at the conserved XhoI site. Sequences from all Y' chromosomal ends (17/32) were analyzed and potential binding sites for Tbf1 and Reb1 proteins determined. Position of ACS is included. *, incomplete sequencing data available at the very distal tip, the end of sequencing data is indicated by the end of the horizontal line. **b** Distances between MNase-sensitive sites identified in Fig. 1d. A size greater than 146 bp is compatible with positioning of a nucleosome, indicated by a dotted line. **c, d** Efficiency of cleavage by MNase-fused proteins on whole genomic DNA. Ethidium bromide staining of XhoI digested genomic DNA - related to Fig. 1c in **c;** and to Fig. 1b in **d**. Time of MNase activity in minutes and MNase-fused proteins are indicated on top of gels. Mono-, di-, tri-, tetra- refer to DNA sizes consistent with mono-, di- tri- and tetra-nucleosomes. M: DNA size marker.

Fig. S2

Chromatin organization of the terminal TEL03L X element. Top: schematic drawing of XY' telomeres (left) and X-only telomeres (right) with positions of XhoI sites. The TG-repeat specific probe used is represented by a solid black line. TRF: Terminal Restriction Fragment. Bottom, autoradiograms of the blots shown in Figs. 1b and 1c as re-hybridized to the TG-repeat specific probe.

Fig. S3

Chromatin organization of the terminal TEL06R X element. a Features identified on all terminal X elements, with respect to their start site. Sequences from X elements of X-only chromosomal ends were analyzed and potential binding sites of Tbf1 and Reb1 determined. Position of ACS is included. The end of X element is indicated by the end of the horizontal line. **b** Analysis of distances between MNase-sensitive sites. Size greater than 146 bp is compatible with positioning of a nucleosome, displayed by a dotted line. **c** related to Fig. 3c and 2b, plot profile of indicated strains. Y axis: Distance (cm), X axis: Intensity signal by pixels (% of total signal). (M) profiles are marker lanes.

Fig. S4

Chromatin organization of subtelomeric elements is independent of telomere length. a Related to Fig. 4a; **b** related to Fig. 4b.; plot profiles of signal intensities obtained with *YKU80* and *yku80Δ* strains. Y axis: Distance (cm), X axis: Intensity signal by pixels (% of total signal). Telomeric area analyzed is indicated on top. Note that the shift to smaller size of the full TRF peak in the *yku80Δ* cells (see gray lines vs black lines) is fully expected as it corresponds to the short telomeric repeat tracts left in *yku80Δ* cells.

Fig. S5

Chromatin organization of X element from XY' chromosomal ends. a Features identified on all X-elements of XY' junctions. Location of the ACS is included. The end of the X element is indicated by the end of the horizontal line. **b** Comparison of cut-sites is plotted with respect to a terminal X (TEL03L) versus an X of an XY' junction (TEL05R). Location of features (ACS, Abf1, Rap1, Tbf1 and Reb1 potential binding sites) are included. **c** Schematic drawing of TEL16R with the position of BamHI and XhoI sites. TEL16R specific probe used in **d** is represented by a solid black line. AF: Analyzed Fragment. **d** *In vivo* ChEC experiments with GBD-MN, NLS-MN and MN-

RAP1 analyzed on X element from TEL16R. Southern blot with BamHI and XhoI-digested genomic DNA hybridized to the TEL16R specific probe. Time of MNase activity in minutes is indicated on top of gels. Arrowheads with solid line indicate detectable cutting common to all MNase-fused proteins. **e** Location of MNase-sensitive sites on the TEL16R XY' junction. Position of features (ACS, Abf1, Rap1, Tbf1 and Reb1 potential binding sites) is included.

Fig. S6

No evidence of a stable telomere foldback structure by *in vivo* ChEC. a Same *in vivo* ChEC as in Fig. 5b on the PvuI fragment of TEL05R obtained with Yku70-MN from *SIR4* (MVY221) and *sir4Δ* (MVL047) strains. **b** Analysis of Reb1 binding on TEL08L from SLIM-ChIP (short-fragment-enriched, low-input, indexed MNase ChIP) previously published (Gutin et al. 2018) **c** Potential binding sites of Rap1 on X-only chromosomal ends. Sequences from X-only chromosomal ends were analyzed and potential binding sites of Rap1 determined. **d** Same as in **c** but analysis was done on the telomere distal X elements of XY' chromosomal ends. Note that the clusters of Rap1 binding sites in the XY'-junction of the TEL13L, TEL12R, TEL14L, TEL12L, TEL06L, TEL04R and TEL08R are due to remaining telomeric TG_{1-3} sequences between the X and Y' elements.

Reference

Gutin, Jenia, Ronen Sadeh, Nitzan Bodenheimer, Daphna Joseph-Strauss, Avital Klein-Brill, Adi Alajem, Oren Ram, and Nir Friedman. 2018. "Fine-Resolution Mapping of TF Binding and Chromatin Interactions." *Cell Reports* 22 (10): 2797–2807. https://doi.org/10.1016/j.celrep.2018.02.052.

Fig. S1

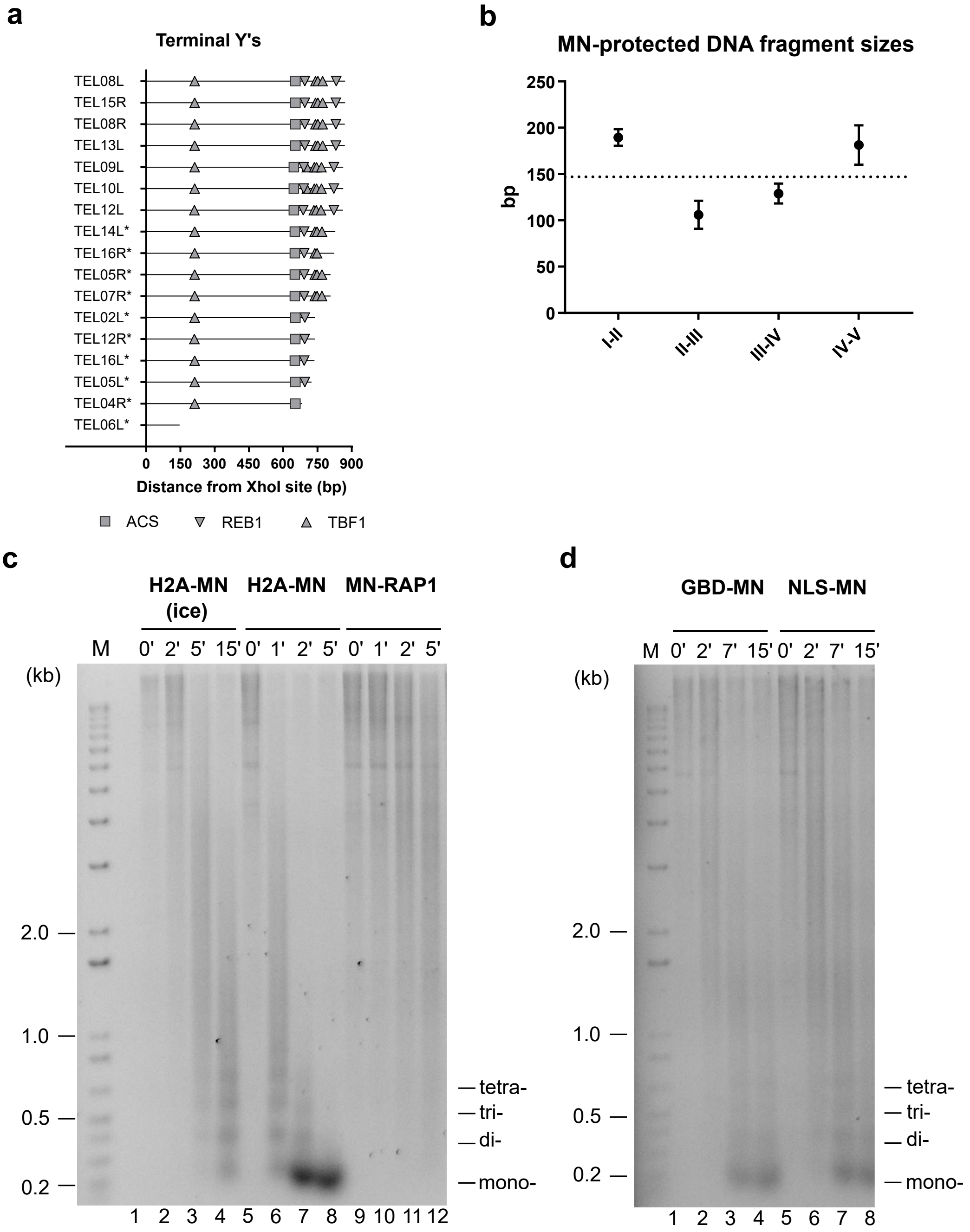
a
Terminal Y's
TEL08L
TEL15R
TEL08R
TEL13L
TEL09L
TEL10L
TEL12L
TEL14L*
TEL16R*
TEL05R*
TEL07R*
TEL02L*
TEL12R*
TEL16L*
TEL05L*
TEL04R*
TEL06L*
0 150 300 450 600 750 900
Distance from XhoI site (bp)
ACS REB1 TBF1
b
MN-protected DNA fragment sizes
bp
250 200 150 100 50 0
I-II II-III III-IV IV-V
c
H2A-MN (ice) H2A-MN MN-RAP1
M 0' 2' 5' 15' 0' 1' 2' 5' 0' 1' 2' 5'
(kb)
2.0
1.0
0.5
0.2
tetra-
tri-
di-
mono-
1 2 3 4 5 6 7 8 9 10 11 12
d
GBD-MN NLS-MN
M 0' 2' 7' 15' 0' 2' 7' 15'
(kb)
2.0
1.0
0.5
0.2
tetra-
tri-
di-
mono-
1 2 3 4 5 6 7 8

Fig. S2

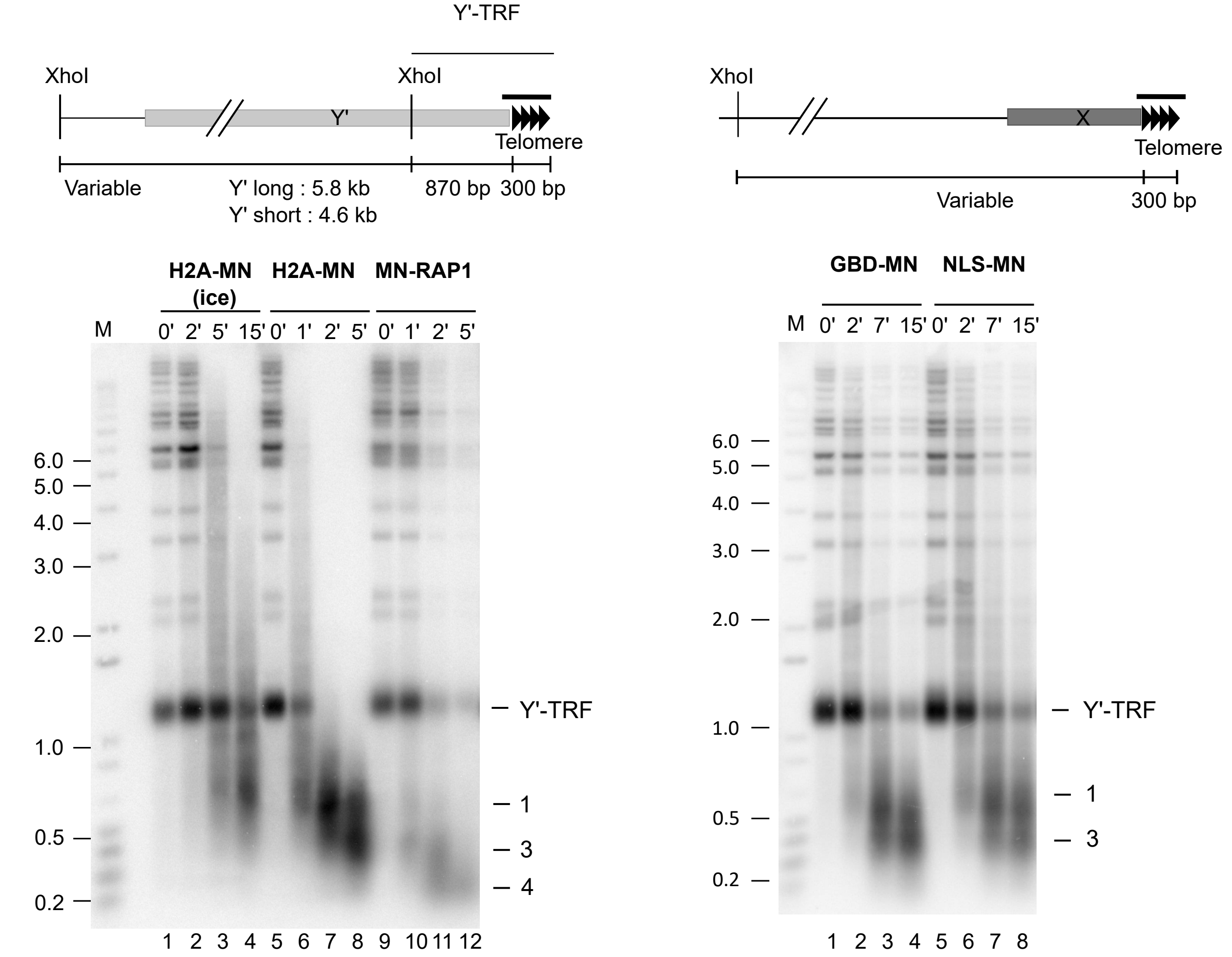

Y'-TRF
XhoI
XhoI
Y'
Telomere
Variable
Y' long : 5.8 kb
Y' short : 4.6 kb
870 bp
300 bp
XhoI
X
Telomere
Variable
300 bp
H2A-MN (ice)
H2A-MN
MN-RAP1
M
0' 2' 5' 15' 0' 1' 2' 5' 0' 1' 2' 5'
6.0
5.0
4.0
3.0
2.0
1.0
0.5
0.2
Y'-TRF
1
3
4
1 2 3 4 5 6 7 8 9 10 11 12
GBD-MN
NLS-MN
M
0' 2' 7' 15' 0' 2' 7' 15'
6.0
5.0
4.0
3.0
2.0
1.0
0.5
0.2
Y'-TRF
1
3
1 2 3 4 5 6 7 8

Fig. S3

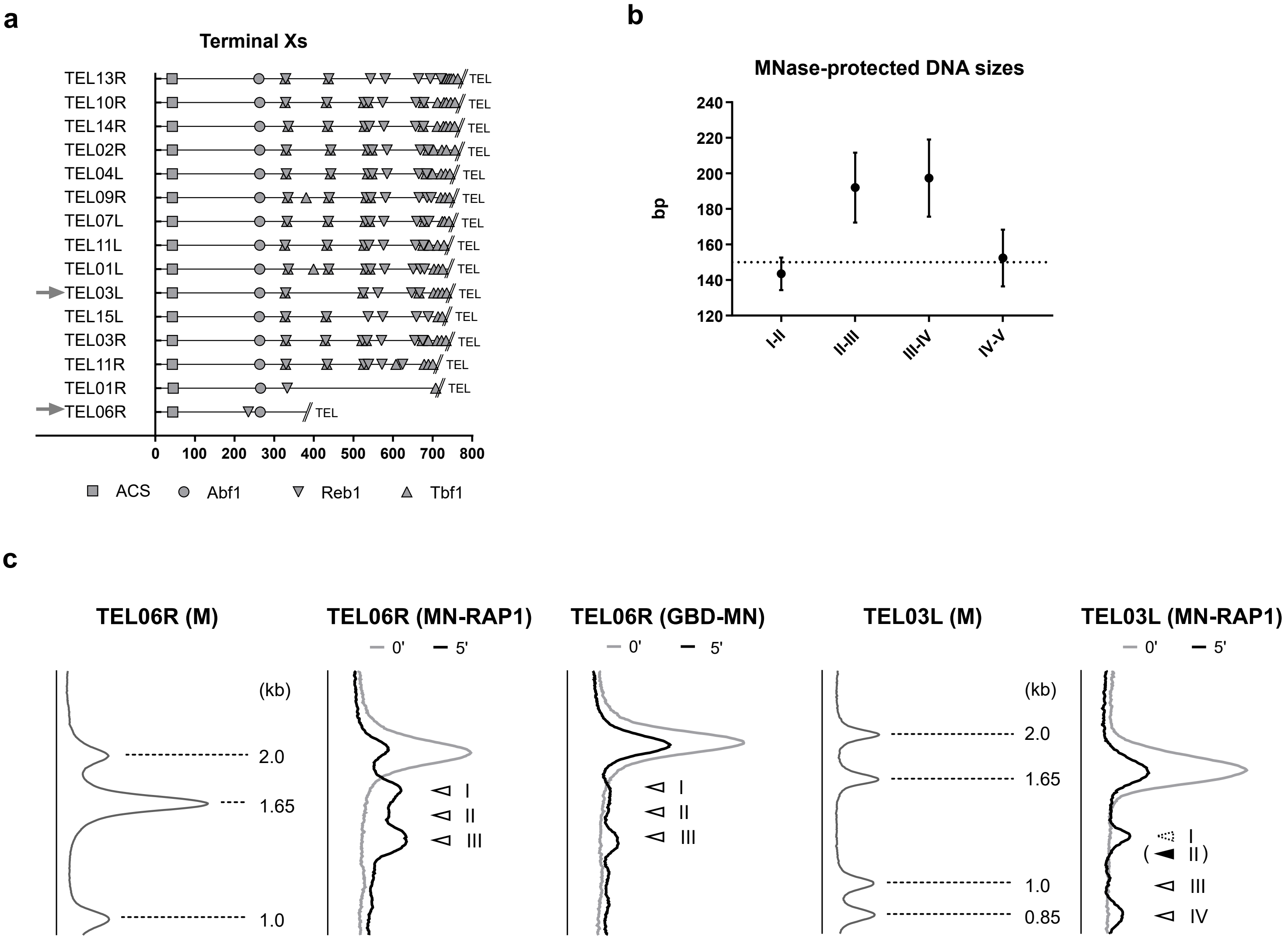
a
Terminal Xs
TEL13R
TEL10R
TEL14R
TEL02R
TEL04L
TEL09R
TEL07L
TEL11L
TEL01L
TEL03L
TEL15L
TEL03R
TEL11R
TEL01R
TEL06R
TEL
0
100
200
300
400
500
600
700
800
ACS
Abf1
Reb1
Tbf1
b
MNase-protected DNA sizes
bp
240
220
200
180
160
140
120
I-II
II-III
III-IV
IV-V
c
TEL06R (M)
TEL06R (MN-RAP1)
TEL06R (GBD-MN)
TEL03L (M)
TEL03L (MN-RAP1)
0'
5'
(kb)
2.0
1.65
1.0
0.85
I
II
III
IV

Fig. S4

a

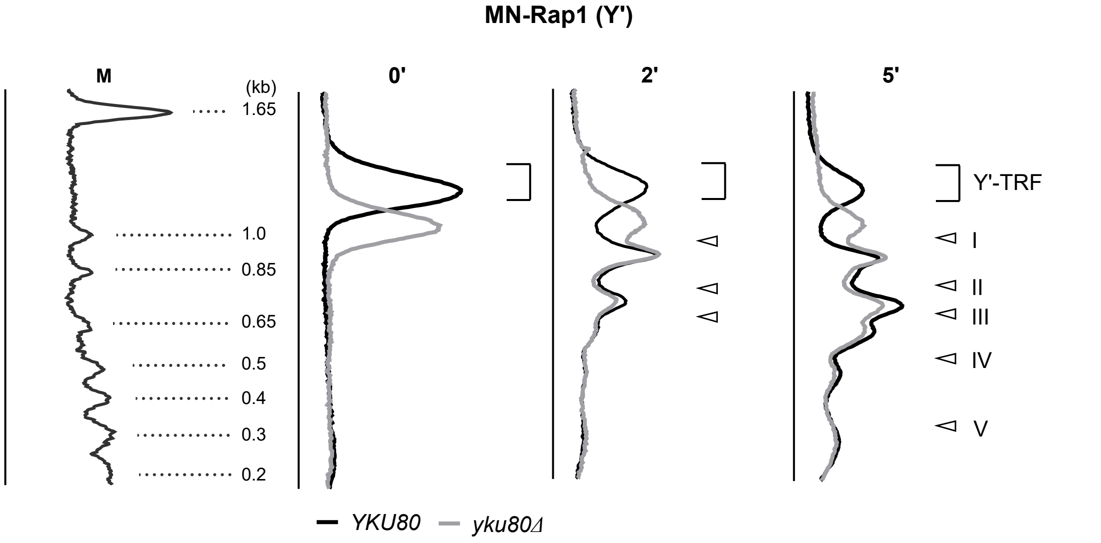
MN-Rap1 (Y')
M
0'
2'
5'
(kb)
1.65
1.0
0.85
0.65
0.5
0.4
0.3
0.2
Y'-TRF
I
II
III
IV
V
YKU80
yku80Δ

b

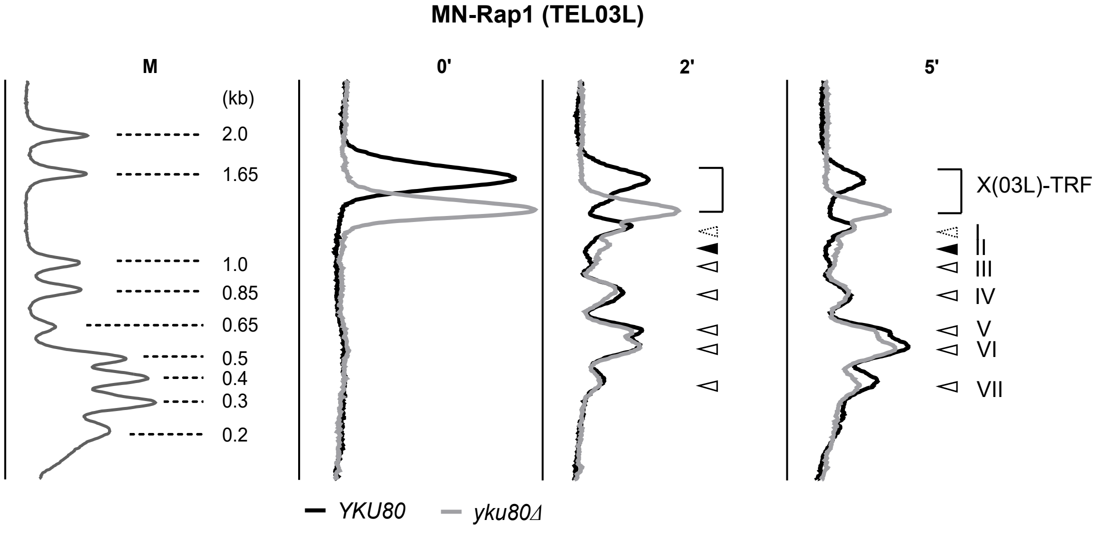
MN-Rap1 (TEL03L)
M
0'
2'
5'
(kb)
2.0
1.65
1.0
0.85
0.65
0.5
0.4
0.3
0.2
X(03L)-TRF
I
II
III
IV
V
VI
VII
YKU80
yku80Δ

Fig. S5

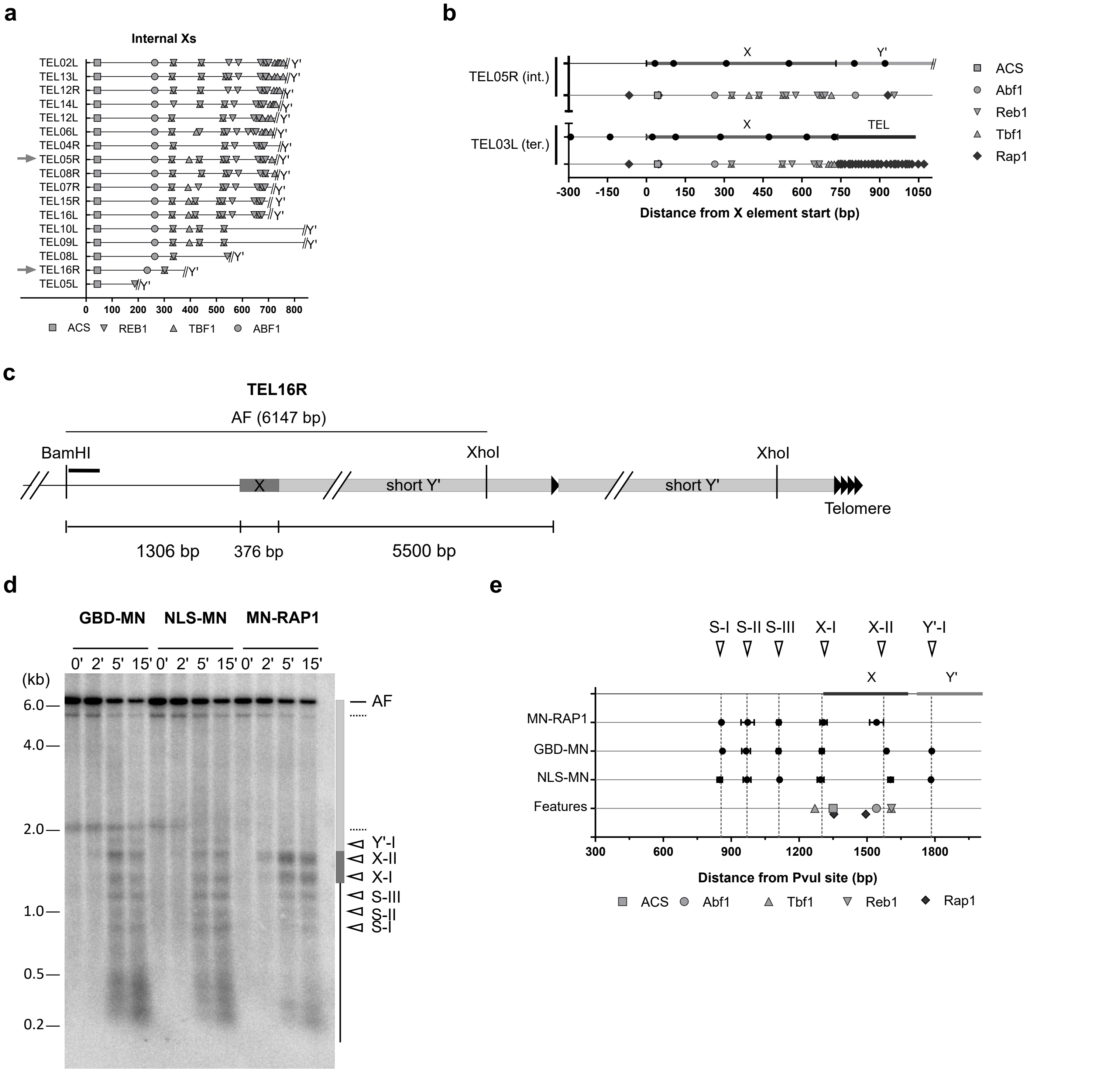
a
Internal Xs
TEL02L
TEL13L
TEL12R
TEL14L
TEL12L
TEL06L
TEL04R
TEL05R
TEL08R
TEL07R
TEL15R
TEL16L
TEL10L
TEL09L
TEL08L
TEL16R
TEL05L
Y'
0 100 200 300 400 500 600 700 800
ACS REB1 TBF1 ABF1
b
TEL05R (int.)
TEL03L (ter.)
X
Y'
TEL
-300 -150 0 150 300 450 600 750 900 1050
Distance from X element start (bp)
ACS
Abf1
Reb1
Tbf1
Rap1
c
TEL16R
AF (6147 bp)
BamHI
XhoI
XhoI
X
short Y'
short Y'
Telomere
1306 bp
376 bp
5500 bp
d
GBD-MN
NLS-MN
MN-RAP1
0' 2' 5' 15' 0' 2' 5' 15' 0' 2' 5' 15'
(kb)
6.0
4.0
2.0
1.0
0.5
0.2
AF
Y'-I
X-II
X-I
S-III
S-II
S-I
e
S-I S-II S-III X-I X-II Y'-I
X
Y'
MN-RAP1
GBD-MN
NLS-MN
Features
300 600 900 1200 1500 1800
Distance from PvuI site (bp)
ACS Abf1 Tbf1 Reb1 Rap1

Fig. S6

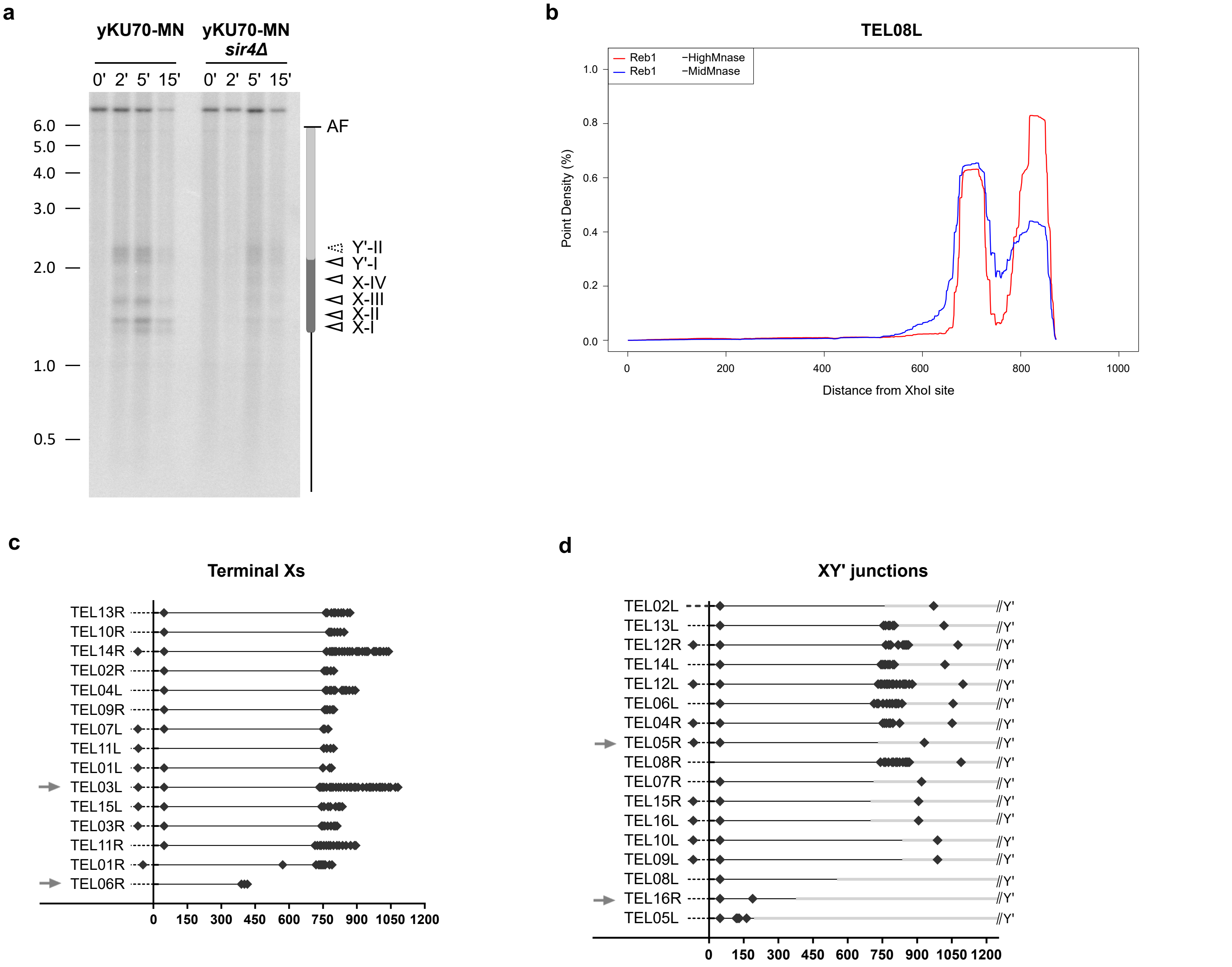

a
yKU70-MN
yKU70-MN sir4Δ
0' 2' 5' 15'
0' 2' 5' 15'
6.0
5.0
4.0
3.0
2.0
1.0
0.5
AF
Y'-II
Y'-I
X-IV
X-III
X-II
X-I
b
TEL08L
Reb1 -HighMnase
Reb1 -MidMnase
Point Density (%)
1.0
0.8
0.6
0.4
0.2
0.0
0
200
400
600
800
1000
Distance from XhoI site
c
Terminal Xs
TEL13R
TEL10R
TEL14R
TEL02R
TEL04L
TEL09R
TEL07L
TEL11L
TEL01L
TEL03L
TEL15L
TEL03R
TEL11R
TEL01R
TEL06R
0 150 300 450 600 750 900 1050 1200
d
XY' junctions
TEL02L
TEL13L
TEL12R
TEL14L
TEL12L
TEL06L
TEL04R
TEL05R
TEL08R
TEL07R
TEL15R
TEL16L
TEL10L
TEL09L
TEL08L
TEL16R
TEL05L
//Y'
0 150 300 450 600 750 900 1050 1200
